# New Lignanamides with Antioxidant and Anti-Inflammatory Activities Screened Out and Identified from *Warburgia ugandensis* Combining Affinity Ultrafiltration LC-MS with SOD and XOD Enzymes

**DOI:** 10.3390/antiox10030370

**Published:** 2021-03-01

**Authors:** Xiao-Cui Zhuang, Gui-Lin Chen, Ye Liu, Yong-Li Zhang, Ming-Quan Guo

**Affiliations:** 1 Key Laboratory of Plant Germplasm Enhancement and Specialty Agriculture, Wuhan Botanical Garden, Chinese Academy of Sciences, Wuhan 430074, China; zhuangxiaocui@yxnu.edu.cn (X.-C.Z.); glchen@wbgcas.cn (G.-L.C.); liuye@wbgcas.cn (Y.L.); zhangyongli@wbgcas.cn (Y.-L.Z.); 2 University of Chinese Academy of Sciences, Beijing 100049, China; 3 Sino-Africa Joint Research Center, Chinese Academy of Sciences, Wuhan 430074, China; 4 Innovation Academy for Drug Discovery and Development, Chinese Academy of Sciences, Shanghai 201203, China; 5 School of Chemical Biology and Environment, Yuxi Normal University, Yuxi 653100, China

**Keywords:** *Warburgia ugandensis*, superoxide dismutase, xanthine oxidase, cyclooxygenase-2, ultrafiltration, antioxidant, anti-inflammatory, lignanamides

## Abstract

*Warburgia ugandensis*, also known as “green heart,” is widely used for the treatment of various diseases as a traditional ethnomedicinal plant in local communities in Africa. In this work, 9 and 12 potential superoxide dismutase (SOD) and xanthine oxidase (XOD) ligands from *W. ugandensis* were quickly screened out by combining SOD and XOD affinity ultrafiltration with LC-MS, respectively. In this way, four new lignanamides (compounds **11**–**14**) and one new macrocyclic glycoside (compound **5**), along with three known compounds (compounds **1**, **3**, and **7**), were isolated and identified firstly in this species. The structures of the new compounds were elucidated by spectroscopic analysis, including NMR and UPLC-QTOF-MS/MS. Among these compounds, compound **14** showed the highest 1,1-diphenyl-2-picrylhydrazyl (DPPH), 2,2′-azinobis-(3-ethylbenzthiazoline)-6-sulfonic acid (ABTS) radical scavenging activities, and total ferric-reducing antioxidant power (FRAP) with IC_50_ values of 6.405 ± 0.362 µM, 5.381 ± 0.092 µM, and 17.488 ± 1.625 mmol TE/g, respectively. Moreover, compound **14** displayed the highest inhibitory activity on cyclooxygenase-2 (COX-2) with IC_50_ value of 0.123 ± 0.004 µM, and the ranking order of other compounds’ IC_50_ values was **13** > **11** > **7** > **1** > **12**. The present study suggested that lignanamides might represent interesting new characteristic functional components of *W. ugandensis* to exert remarkable antioxidant and anti-inflammatory activities. Moreover, compound **14**, a new arylnaphthalene lignanamide, would be a highly potential natural antioxidant and anti-inflammatory agent from *W. ugandensis*.

## 1. Introduction

*Warburgia ugandensis*, a kind of evergreen tree with a distinctive aromatic smell, belongs to the Canellaceae family, and is mainly distributed in many countries in eastern and southern Africa, with a few in India [1]. As a well-known traditional medicinal plant in local communities in Africa, this species has long been widely used for the treatment of headache, backache, stomachache, toothache, sore throat, asthma, sexually transmitted diseases, rheumatoid arthritis, measles, loss of appetite, constipation, diarrhea, parasites, skin diseases, cough, cold, fever, seizure, tuberculosis, pneumonia and malaria by different methods of preparation and administration [2,3]. To date, more than 80 chemical components have been found in *W. ugandensis*, mainly including sesquiterpenes [4,5,6,7,8], flavonoids and their glycosides [9], fatty acids [10], etc. Bioactivities of different solvent extracts or isolated compounds are mainly manifested in antioxidant [11], anti-inflammatory [12], antibacterial [13], antifungal [5], antiviral [14], and antiparasitic activities and cytotoxicity [15].

Recent studies suggested that oxidative stress, mainly including superoxide anion radical (O_2_^•−^), hydroxyl radical (∙OH), and hydrogen peroxide (H_2_O_2_), could cause damage to multiple organs of the human body [16]. For example, xanthine oxidase (XOD) is a crucial flavoprotein enzyme, which participates in the catalysis of the oxidative hydroxylation of hypoxanthine and xanthine to generate uric acid and O_2_^•−^ [17]. Moreover, produced O_2_^•−^ contributes to the pathogenesis of mental disorders [18], neurodegenerative diseases [19], cardiovascular diseases [20], chronic obstructive pulmonary diseases (COPD) [21], gouts, hyperuricemias, hepatitis, carcinogenesis, and aging [22]. In contrast, superoxide dismutase (SOD) is an indispensable enzyme which catalyzes O_2_^•−^ to H_2_O_2_ and oxygen (O_2_). Moreover, H_2_O_2_ can be decomposed to a hydroxy radical (∙OH) and hydroxide ion (OH^−^) in the presence of transition metal ions. Hence, SOD plays an important role in the balance between oxidation and anti-oxidation [23]. Thus far, several reports illustrated that sesquiterpenes from *W. ugandensis* exhibited antioxidant, antiradical [11], and anti-inflammatory activities [12]. However, the indistinctness of its antioxidant and anti-inflammatory clinic practices in local communities in Africa and correlated bioactive phytochemicals are still elusive, and hence, this needs to be further comprehensively addressed for the treatment of oxidation-related diseases.

In this study, 1,1-diphenyl-2-picrylhydrazyl (DPPH), 2,2′-azinobis-(3-ethyl-benzthiazoline)-6-sulfonic acid (ABTS) radical scavenging and ferric ion reducing antioxidant power (FRAP) assays were firstly used to analyze the antioxidant capacities of different stem bark extracts from *W. ugandensis*. Then, SOD- and XOD-based affinity ultrafiltration (UF), combined with high performance liquid chromatography-tandem mass spectrometry (LC-MS/MS), were applied to rapidly screen bioactive components from the most potential fraction (WUE-A4). Finally, eight compounds were isolated and identified from WUE-A4, and their antioxidant and anti-inflammatory activities were tested. For the first time, the present study comprehensively illustrated the notable antioxidant and anti-inflammatory activities and its correlated bioactive phytochemicals of *W. ugandensis*, which provides valuable information to promote the applications of this species as a natural antioxidant and anti-inflammatory agent.

## 2. Materials and Methods

### 2.1. General Experimental Procedures

The UV absorbance was recorded by multifunctional microplate reader (Tecan Infinite M200 PRO, TECAN, Männedorf, Switzerland) and UV-1100 spectrophotometer (Shanghai Mapada Instrument Co., Ltd., Shanghai, China). Thirty kDa (YM-30) and 10 kDa (YM-10) ultrafiltration membranes were purchased from Millipore Co. Ltd. (Bedford, MA, USA). The HPLC-UV/ESI-MS/MS was carried out by Thermo Accela 600 series HPLC connected with a TSQ Quantum Access MAX mass spectrometer (Thermo Fisher Scientific, San Jose, CA, USA). NMR spectra were recorded on a Bruker Avance III 600 MHz (Bruker, Karlsruhe, Germany) with TMS as an internal standard. UPLC-QTOF-MS/MS data were collected by ultra-performance liquid chromatography quadrupole time of flight mass spectrometry (UPLC-QTOF-MS/MS) (Agilent Technologies, Santa Clara, CA, USA) with an ACQUITY UPLC BEH column (50 × 2.1 mm, 1.7 µm, ACQ, Waters Co., Milford, MA, USA). Column chromatography (CC) were undertaken on AB-8 macroporous adsorbent resin (Qingdao Haiyang Chemical Co., Ltd., Qingdao, China), ODS-A-HG (12 nm, S-50 µm, YMC Co., Ltd., Tokyo, Japan), and Sephadex LH-20 (25–100 µm, Pharmacia Fine Chemical Co., Ltd., Uppsala, Sweden). Analytic HPLC was carried out using an Agilent 1220 liquid chromatograph (Agilent Technologies, Santa Clara, CA, USA) with a Waters Symmetry RP-C18 column (4.6 mm × 250 mm, 5 µm). Semipreparative HPLC was carried out using an Agilent 1100 liquid chromatograph (Agilent Technologies, Santa Clara, CA, USA) and a Hanbon NS4205 chromatograph (Hanbon Sci. & Tech., Jiangsu, China), with the columns of a YMC-Pack ODS-A (250 mm × 9.4 mm, i.d., 5 µm) (YMC Co., Ltd., Tokyo, Japan) and an Agilent Eclipse XDB-C18 (250 mm × 9.4 mm, i.d., 5 µm) (Agilent Technologies, Santa Clara, CA, USA). Preparative HPLC was carried out using a Hanbon NS4205 chromatograph (Hanbon Sci. & Tech., Jiangsu, China), with the column of Smuwei C18 (250 mm × 30 mm, i.d., 10 µm) (Hanbon Sci. & Tech., Jiangsu, China). Silica gel plate for thin layer chromatography (TLC) (Qingdao Haiyang Chemical Co., Ltd., Qingdao, China) was used directly for quick separation.

### 2.2. Chemicals and Reagents

6-Hydroxy-2,5,7,8-tetramethylchroman-2-carboxylic acid (Trolox), 1,1-diphenyl-2-picrylhydrazyl (DPPH), 2,2′-azinobis-(3-ethylbenzthiazoline-6-sulfonic acid) (ABTS), 1,3,5-tri(2-pyridyl)-2,4,6-triazine (TPTZ). Millipore membranes (0.22 µm) were provided by Tianjin Jinteng Experiment Equipment Co., Ltd. (Tianjin, China). Ultrapure water was produced by the ultrapure water polishing system (Nanjing Yeap Esselte Technology Development Co., Nanjing, China). Analytically pure chemicals and solvents, such as acetonitrile (ACN), methanol (MeOH), ethanol (EtOH), dimethyl sulfoxide (DMSO), petroleum ether, ethyl acetate, *n*-butanol, formic acid, concentrated sulfuric acid, sodium hydroxide, tris(hydroxymethyl)aminomethane (Tris), hydrochloric acid (HCl), ferric chloride, and potassium ferricyanide, were purchased from Sinopharm Chemical Reagent Co., Ltd. (Shanghai, China). Chromatographically pure acetonitrile and methanol were purchased from Tedia Company Int., (Fairland, USA). SOD and XOD were obtained from Shanghai yuanye Bio-Technology Co., Ltd. (Shanghai, China). COX-2 (human) inhibitor screening assay kits (No. S0168) were purchased from Beyotime Biotechnology (Shanghai, China).

### 2.3. Plant Materials

The stem barks of *W. ugandensis* were collected in June 2018 from Narok County (latitude: −1.2408°, longitude: 35.7356°, altitude: 1851 m), Kenya, and authenticated by Prof. Guangwan Hu, a taxonomist of Wuhan Botanical Garden, Chinese Academy of Sciences. A voucher specimen (No. WBG-ZWHX 201808001) was deposited in the herbarium of the Key Laboratory of Plant Germplasm Enhancement and Specialty Agriculture.

### 2.4. Preparation of Samples

#### 2.4.1. Preparation of Extractions and Fractions

Air-dried powders of stem barks from *W. ugandensis* (4.8 kg) were extracted three times with 95% ethanol (30 L × 3) at room temperature for 6 days, and then concentrated to obtain crude extracts (WUZ, 391.4 g). The crude extracts were dispersed in ultrapure water (1 L) to gain a homogeneous mixture, which was successively partitioned three times with equal volumes of petroleum ether, ethyl acetate, and *n*-butanol to get four fractions: petroleum ether fraction (WUP), ethyl acetate fraction (WUE), *n*-butanol fraction (WUN), and H_2_O fraction (WUH). The ethyl acetate fraction (91.1 g) was adsorbed by a macroporous adsorbent resin (AB-8) column, and then washed using gradient elution with EtOH-H_2_O. The resulting fractions were combined based on TLC analysis to give eight fractions for further experiments: Fr. WUE-A–Fr. WUE-H. Fr. WUE-A (22.6 g) was separated by reverse phase silica gel (ODS-A-HG) CC using gradient elution with a solvent system of MeOH-H_2_O (10–55%) to produce seven subfractions: WUE-A1–WUE-A7.

#### 2.4.2. Isolation of Pure Compounds

WUE-A4 (802.1 mg) was subjected to Sephadex LH-20 CC and eluted with 70% MeOH-H_2_O to get five subfractions: A41–A45. Subfraction A43 (140.4 mg) was purified by semipreparative HPLC with 34% MeOH-H_2_O (H_2_O contains 0.1% formic acid, flow rate 3.0 mL/min) to get two subfractions: A431–A432. Subfraction A432 (68.2 mg) was purified by semipreparative HPLC with 17% ACN-H_2_O (H_2_O contains 0.1% formic acid, flow rate 2.5 mL/min) to yield compound **13** (10.4 mg; Rt = 52 min). Subfraction A44 (182.4 mg) was purified by semipreparative HPLC with 10% ACN-H_2_O (flow rate 2.5 mL/min) to yield compound **1** (6.4 mg; Rt = 20 min), compound **3** (9.3 mg; Rt = 25 min), and compound **5** (2.2 mg; Rt = 27 min). Subfraction A45 (162.3 mg) was purified by semipreparative HPLC with 32% MeOH-H_2_O (H_2_O contains 0.1% formic acid, flow rate 3.0 mL/min) to obtain compound **14** (19.1 mg; Rt = 40 min) and subfractions: A451–A453 and A455–A456. Subfraction A452 (67.3 mg) was purified by semipreparative HPLC with 18% ACN-H_2_O (flow rate 2.5 mL/min) to yield compound **7** (3.0 mg; Rt = 35 min), compound **11** (3.6 mg; Rt = 58 min), and compound **12** (6.6 mg; Rt = 61 min). Finally, these pure compounds isolated were completely dried and dissolved in methanol-*d*_4_ or DMSO-*d*_6_, and their ^1^H NMR, ^13^C NMR, and 2D NMR spectra were measured.

### 2.5. In Vitro Antioxidant Assays of Samples

#### 2.5.1. DPPH Assays

The DPPH free radical scavenging activities of extracts and pure compounds from stem barks of *W. ugandensis* were tested according to previous studies with slight modifications [24,25]. Firstly, 10 µL solutions of the samples, appropriately diluted with MeOH, were homogeneously mixed with 190 µL solution of DPPH (0.1 mM in MeOH) in a 96-well plate. Secondly, the mixtures were gently shaken and put at room temperature for 30 min in the darkness. Finally, the absorbance was recorded at 517 nm with multifunctional microplate reader. Trolox was used as positive control, and MeOH was used as blank control. Each of the samples and controls were tested in triplicate (n = 3). The antioxidant activities of DPPH were expressed as IC_50_ (concentration of samples caused 50% inhibition) and TEAC values. The TEAC values were calculated from the standard curve of Trolox and expressed as millimoles of Trolox equivalents per gram of sample (mmol TE/g). The inhibition of the DPPH radical was calculated according to the following formula:

DPPH scavenging activity (%) = (1 − absorbance of sample/absorbance of blank control) × 100,(1)

where DPPH scavenging activities (%) were plotted against the concentration of samples to obtain the IC_50_.

#### 2.5.2. ABTS Assays

The ABTS free radical scavenging activities of extracts and pure compounds from stem barks of *W. ugandensis* were tested according to previous studies with slight modifications [24,25]. ABTS^+^ solution was prepared by mixing equal volumes of potassium persulfate (4.9 mM in H_2_O) and ABTS (7.0 mM in H_2_O) and then incubated for 12 h in the darkness. The prepared ABTS^+^ solution was diluted with MeOH to ensure an absorbance value about 0.700 ± 0.005 at 734 nm. Firstly, 10 µL of appropriately diluted solution of samples was added to the 190 µL ABTS^+^ solution in 96-well plate. Secondly, the mixtures were gently shaken and put at room temperature for 30 min in the darkness. Finally, the absorbance was recorded at 734 nm with multifunctional microplate reader. Trolox was used as positive control, and MeOH was used as blank control. Samples and controls were tested in triplicate (n = 3). The antioxidant activities of ABTS were expressed as IC_50_ and TEAC values. The inhibition of the ABTS radicals was calculated according to the following formula:

ABTS scavenging activity (%) = (1 − absorbance of sample/absorbance of blank control) × 100,(2)

where ABTS scavenging activities (%) were plotted against the concentration of samples to acquire the IC_50_.

#### 2.5.3. FRAP Assays

The ferric reducing antioxidant power assays on different extracts and pure compounds of stem barks from *W. ugandensis* were tested according to previous studies with slight modifications [24,25]. Firstly, freshly prepared FRAP reagent (Fe^3+^-TPTZ solution) was composed of FeCl_3_∙6H_2_O (20 mM in H_2_O), TPTZ (10 mM in 40 mM HCl), and acetate buffer (300 mM, pH = 3.6) at a ratio of 1:1:10 (*v*/*v*/*v*), then incubated at 37 °C after preparation, and used within 1–2 h. Secondly, 10 µL of appropriately diluted solution of samples was added to the 190 µL freshly prepared FRAP reagent in 96-well plate. Thirdly, the mixtures were gently shaken and put at 37 °C for 10 min. Finally, the absorbance was recorded at 593 nm with multifunctional microplate reader. Trolox was used as a positive control, and MeOH was used as blank control. Each of the samples and controls were tested in triplicate (n = 3). The antioxidant activities of FRAP were expressed as FRAP values and TEAC values. The FRAP values were calculated and expressed as millimoles of Fe^2+^ equivalents per gram of sample (mmol Fe^2+^/g) based on a calibration curve plotted using FeSO_4_∙7H_2_O as standard at a concentration ranging from 0.0185 to 1.5 mM.

### 2.6. In Vitro COX-2 Inhibitory Assays

COX-2 inhibition assay in vitro was performed using COX-2 (human) inhibitor screening assay kits according to the manufacturer’s instructions to evaluate the COX-2 inhibition activity of compounds and verify the results of UF-LC-MS/MS [26,27]. Firstly, samples were dissolved in DMSO, and prepared into a series of solutions with different concentrations. COX-2 cofactor working solution, COX-2 working solution, COX-2 probe, and COX-2 substrate were prepared according to manufacturer’s instructions, and then diluted 10 times with COX-2 assay buffer, respectively. Secondly, 150 µL Tris-HCl (pH = 7.8), 10 µL COX-2 cofactor working solution, 10 µL COX-2 working solution, and 10 µL sample solution were sequentially added in the 96-well black plates, mixed and incubated at 37 °C for 10 min. The COX-2 working solution of the blank control group was replaced with an equal volume of COX-2 assay buffer, and the sample was replaced with an equal volume of DMSO; the sample of the 100% enzyme activity control group was replaced with an equal volume of DMSO. Thirdly, 10 µL COX-2 probe was added into each well. Finally, 10 µL of COX-2 substrate was quickly added into each well, and incubated at 37 °C in the darkness for 5 min, and followed by the fluorescence measurement. The excitation and emission wavelengths were 560 nm and 590 nm, respectively. Indomethacin was set as a positive control. The experiments were performed in triplicate. The COX-2 inhibitory activity was expressed as IC_50_. The inhibition of COX-2 was calculated according to the following formula:

COX-2 inhibitory activity (%) = (RFU_100%Enzyme_ − RFU_Sample_)/ (RFU_100%Enzyme_ − RFU_Blank_) × 100, (3)

where COX-2 inhibitory activity (%) was plotted against the concentration of samples to acquire the IC_50_. RFU_100%Enzyme_, relative fluorescence unit of 100% enzyme control group; RFU_Sample_, relative fluorescence unit of sample; RFU_Blank_, relative fluorescence unit of blank group.

### 2.7. Screening and Identification of the Potential Ligands of SOD and XOD with UF-LC-MS/MS

#### 2.7.1. Affinity Ultrafiltration with SOD and XOD

Potential bioactive components which had a high binding affinity to SOD and XOD were screened by affinity ultrafiltration. The experimental conditions were proposed according to previous relevant studies [27,28]. Briefly, the optimized experiment was composed of three steps. Incubation was the first step, where 100 μL WUE-A4 (10.0 mg/mL), 10 µL SOD (2 U) or XOD (2 U), and 90 µL Tris-HCl (pH = 7.8) were mixed well in 1.5 mL EP tubes, and then incubated at 37 °C in the dark for 1 h. Meanwhile, the incubation operations of inactivated enzymes (inactivated enzymes were obtained by heating enzymes in a 99 °C water bath for 10 min) were the same as the active enzymes. Adsorption was the second step, where the incubated solutions were transferred to ultrafiltration tubes with 30 KDa (for SOD) or 10 KDa (for XOD) ultrafiltration membranes and then centrifuged at 10,000 rpm for 10 min at 25 °C. Simultaneously, components which had no binding affinity to enzymes were washed out. Immediately, 300 µL of Tris-HCl solution (pH = 7.8) was added to the ultrafiltration tubes and centrifuged at 10,000 rpm for 5 min at 25 °C to remove the potential unbound components. Desorption was the third step, where 300 µL of 90% (*v*/*v*) MeOH-H_2_O was added and incubated for 10 min at room temperature, and the mixed solutions were centrifuged at 10,000 rpm for another 10 min at 25 °C. After that, the desorption process was repeated two times to release those components with specific bindings to SOD or XOD from the enzyme-ligand complexes. Finally, those ultrafiltrates were dried and reconstituted in 50 µL MeOH for the HPLC-UV/ESI-MS/MS analysis.

#### 2.7.2. HPLC-UV/ESI-MS/MS Analysis

HPLC-UV/ESI-MS/MS was carried out to characterize the components in WUE-A4 before and after ultrafiltration by using a Thermo Accela 600 series HPLC connected with a TSQ Quantum Access MAX mass spectrometer (Thermo Fisher Scientific, San Jose, CA, USA). A Waters Symmetry RP-C18 column (4.6 × 250 mm, 5 µm) was used to perform chromatographic analysis at 30 °C, and the mobile phase consisted of H_2_O with 0.1% formic acid (A) and ACN (B). The optimized HPLC elution procedures were as follows: 0–15 min, 17% B; 15–40 min, 17–30% B, 40–42 min: 30–56% B. The flow rate was 0.8 mL/min, the injection volume was 10 µL, and the HPLC-UV chromatograms were detected at a wavelength of 254 nm. The negative ion modes were applied to obtained ESI-MS/MS data. Moreover, the parameters of instrument were set as follows: the vaporizer temperature was 350 °C, the capillary temperature was 250 °C, the spray voltage was 3000 V, the cone voltage energy was 40 V, the collision energy was 10 V, the sheath gas pressure was 40 psi, the aux gas pressure was 10 psi, the drying gas flow rate was 6.0 L/min, and the mass range was set from 50 to 1100 (*m/z*) in the full-scan mode. Finally, the Thermo Xcalibur ChemStation (Thermo Fisher Scientific) was used for data acquisition and analysis.

### 2.8. Statistical Analysis

All data in this work were expressed as mean ± standard deviation (SD) of triplicate measurements. The percentages of scavenging activities or the inhibition rates were plotted against the sample concentrations (six different concentration gradients in triplicate) to obtain the IC_50_ values, defined as the concentrations of samples necessary to cause 50% scavenging or inhibition. Software used for statistical analysis mainly included SPSS 16.0 (SPSS Inc., Chicago, IL, USA), Graphap Prism 5.0 (GraphPad Software Inc., San Diego, CA, USA), Origin 2019b (OriginLab Corporation, Northampton, MA, USA), Chemoffice 18.0 (CambridgeSoft Corp., Cambridge, MA, USA), and MestreNova (Mestrelab Research SL, San Diego, CA, USA).

## 3. Results and Discussion

### 3.1. Antioxidant Activities of Different Extracts of Stem Barks from W. ugandensis

**Figure 1:**
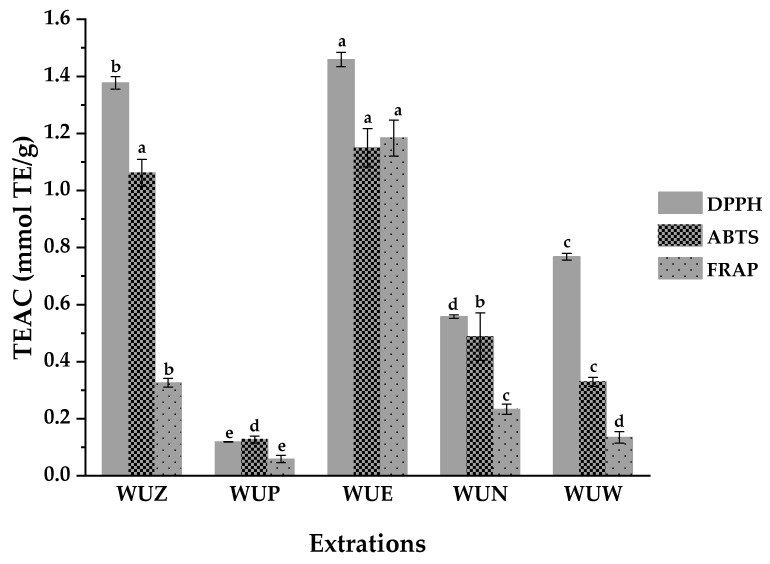
Trolox equivalent antioxidant capacity (TEAC) of different extracts from stem barks of *W. ugandensis* tested by DPPH, ABTS, and FRAP assays. Means labeled by different letters ^(a–e)^ were significantly different at a level of *p* < 0.05 (n = 3) by DMRT (Duncan’s multiple range test).

It is inappropriate to assess the antioxidant activity only by a single method for the reason of the complexity of chemical constituents and their diverse mechanisms of action. Thus, the DPPH, ABTS, and FRAP assays were employed in the present study to assess the antioxidant activities of five extracts of stem barks from *W. ugandensis*, respectively, according to other relevant reports in our laboratory [29,30]. As shown in Table 1, WUE displayed the highest antioxidant activities among these five extracts, with the IC_50_ values of the DPPH radical scavenging activity at 17.800 ± 0.300 µg/mL, ABTS radical scavenging activity at 9.400 ± 0.529 µg/mL, and FRAP at 5.579 ± 0.296 mmol Fe^2+^/g, respectively. Moreover, WUZ also exhibited relatively higher antioxidant activities in comparison with other extracts based on the TEAC assay (Figure 1). Similarly, the 80% ethanol crude extracts of stem barks of *W. salutaris* also exerted potential antioxidative activities [31].

### 3.2. Antioxidant Activities of Different Fractions Eluted from WUE

**Figure 2:**
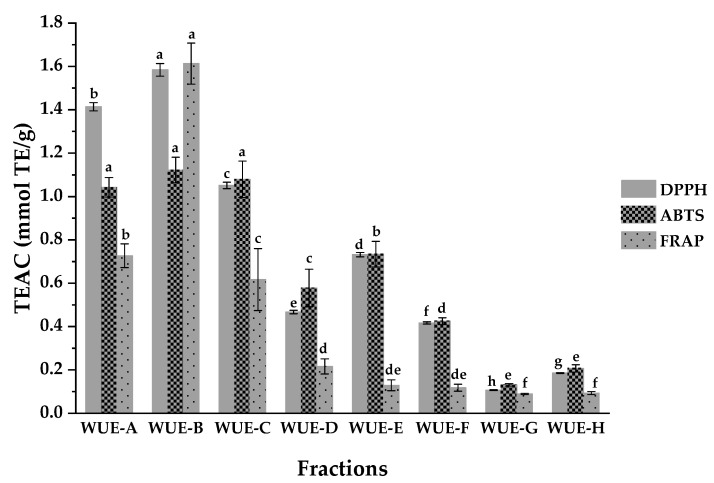
Trolox equivalent antioxidant capacity (TEAC) of different fractions from WUE tested by DPPH, ABTS, and FRAP assays. Means labeled by different letters (^a–h^) were significantly different at a level of *p* < 0.05 (n = 3) by DMRT (Duncan’s multiple range test).

The antioxidant activities of eight fractions obtained from WUE were also tested by three assays to single out the most effective fraction(s). As shown in Table 2 and Figure 2, the results suggested that fraction WUE-B possessed the highest antioxidant activities among these eight fractions, with the IC_50_ values of DPPH, ABTS, and FRAP antioxidant activities at 16.400 ± 0.300 µg/mL, 9.633 ± 0.513 µg/mL, and 7.603 ± 0.446 mmol Fe^2+^/g, respectively. Moreover, WUE-A also displayed relatively higher antioxidant activities in comparison with other fractions according to Table 2. To this end, fractions WUE-B and WUE-A were, hence, systematically explored for the affinity ultrafiltration screening, isolation, and purification of the main bioactive constituents with high antioxidant activities in the subsequent operations.

### 3.3. Screening and Identification of SOD and XOD Ligands by UF-LC-MS/MS

#### 3.3.1. Screening for the Potential SOD and XOD Ligands in WUE-A4

In previous study, colorotane sesquiternepes extracted from *W. ugandensis* were speculated as possible antioxidant and antiradical components by using computational tools [11]. However, no substantial evidences for both its active components and their corresponding targets were explored so far. Since UF-LC-MS/MS could be used to rapidly screen out bioactive chemical components from complex plant extracts depending on the binding properties between the enzymes and their ligands [26,27,28,32], we further employed UF-LC-MS/MS to quickly screen out and identify the potential ligands of SOD and XOD in WUE-A4 from Kenya in this work. As a result, it is firstly revealed that *W. ugandensis* contains components with strong binding affinity to both SOD and XOD.

**Figure 3:**
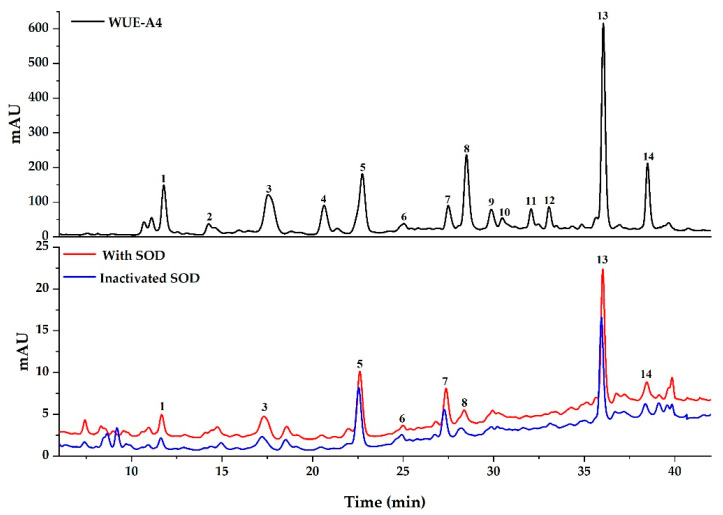
The UF-HPLC-UV chromatograms of WUE-A4 with superoxide dismutase. The black, red, and blue lines represent the HPLC-UV profiles of WUE-A4 without ultrafiltration, with activated and inactivated superoxide dismutase, respectively.

**Figure 4:**
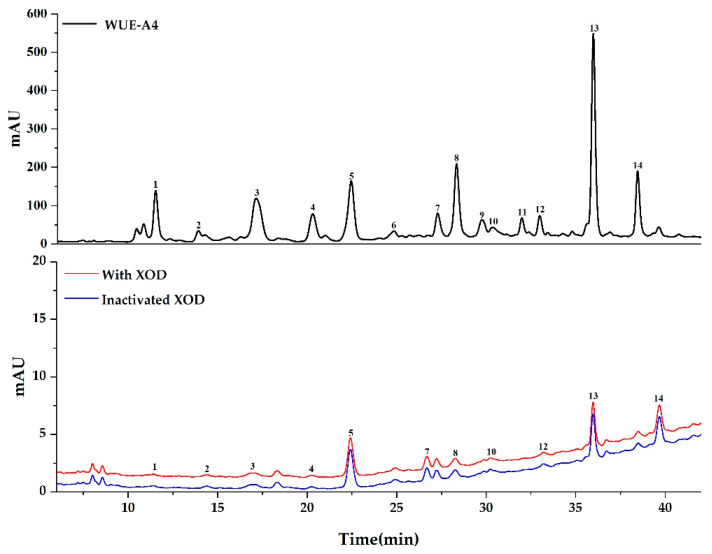
The UF-HPLC-UV chromatograms of WUE-A4 with xanthine oxidase. The black, red, and blue lines represent the HPLC-UV profiles of WUE-A4 without ultrafiltration, with activated xanthine oxidase, and with inactivated xanthine oxidase, respectively.

As shown in Figure 3 and Figure 4, components in WUE-A4 displayed various binding affinities to SOD and XOD, respectively. Based on the diversities of affinity capabilities between ligand-enzyme complexes after incubation, those potential ligands in groups with the active enzymes displayed bigger peak areas than those of with inactivated enzymes. For further evaluation of the affinity binding strength between the potential ligands and the target enzymes, the relative binding affinity (RBA) was employed to compare the variation of the correlated peak areas in the UF-HPLC-UV chromatograms before and after activation [33]. The RBA formula is expressed as RBA = A_active_/A_inactivated_, where the A_active_ and A_inactivated_ represent the peak areas obtained from the WUE-A4 samples with activated and inactivated SOD or XOD, respectively. Table 3 lists the RBAs of the potential ligands in WUE-A4 targeting SOD and XOD. For XOD, peak 14 displayed the highest RBA, with a value of 3.76, followed by peak 8 with a value of 2.35, peak 10 with a value of 2.33, peak 6 with a value of 2.09, peak 2 with a value of 1.76, peak 12 with a value of 1.70, and so on. For SOD, peak 3 exhibited the highest RBA value, with a value of 1.99, followed by peak 1 with a value of 1.93, peak 8 with a value of 1.87, peak 4 with a value of 1.55, peak 14 with a value of 1.51, etc.

As shown in Figure 3, Figure 4, and Table 3, the binding affinities of some bioactive components among the potential bioactive components in WUE-A4 with SOD were consistent with XOD, for example, the RBA values of peaks 8 and 14 were higher than others. In other words, considering the strong affinities of peaks 8 and 14 with both SOD and XOD, it could be assumed that the two components were the main bioactive components in WUE-A4, which were closely related to its noteworthy antioxidant capacity. In addition, for the specific components, the binding affinities of peaks 2, 6, 10, and 12 with XOD were significantly stronger than that of SOD with higher RBA values, while peaks 1, 3, and 4 exhibited stronger binding affinities with SOD than that of XOD with higher RBA values. In this regard, each potential peak (component) in WUE-A4 exerted diverse binding affinities to these two enzymes. However, peaks 9 and 11 exhibited extremely low or no binding affinities to both SOD and XOD, due to no visible chromatographic peaks in Figure 3 and Figure 4 after ultrafiltration screening.

#### 3.3.2. Compounds Isolated and Identified from WUE-A4

**Figure 5:**
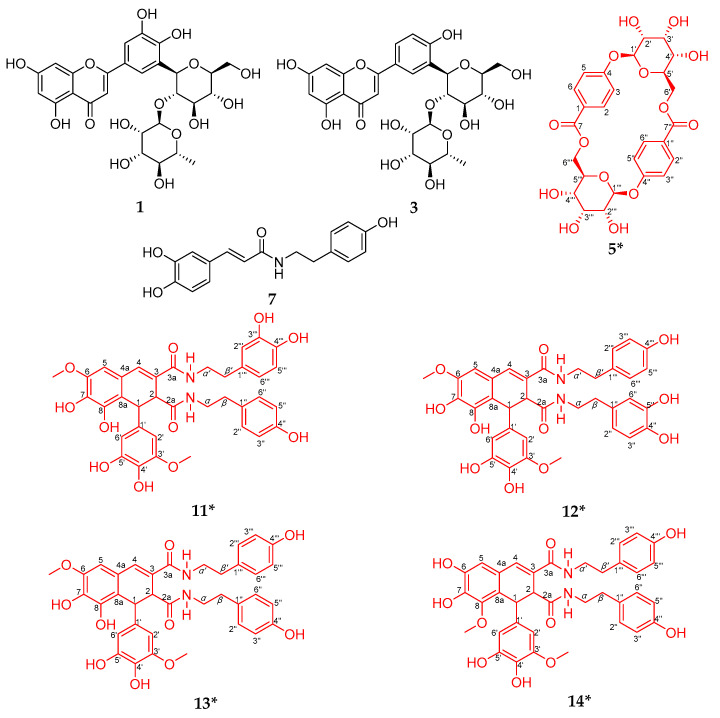
Compounds isolated and identified from WUE-A4. * New compounds; the red structures belong to new compounds.

Nine and 12 components in WUE-A4 showed distinctive binding affinities to SOD and XOD after the affinity ultrafiltration screening, respectively, as shown in Figure 3, Figure 4, and Table 3. These potential ligands were identified and characterized by HPLC-UV/ESI-MS/MS analysis. The mass spectrum data, such as Rt, *m/z*, and representative MS/MS fragments in the negative mode, were listed in Table 3. Theoretically, bioactive components in WUE-A4 could be tentatively identified by characteristic MS/MS fragments as compared to known databases or standards. However, for some unknown or new compounds, the isolation of pure compounds and subsequent structure elucidation should also be done. In this context, eight compounds were isolated from WUE-A4 by using modern separation and purification techniques, including CC, TLC, and HPLC, and then their structures were accurately identified by NMR and UPLC-QTOF-MS/MS (detail compounds data were recorded in Supplementary Materials). Isolation of WUE-A4 resulted in the identification of eight compounds (Figure 5), including four new lignanamides: 1-(3,4-dihydroxy-5-methoxyphenyl)-1,2-dihydroxy-7,8-dihydroxy-*N*-[(3,4-dihydroxyphenyl)-ethyl]-*N*′-[(4-hydroxyphenyl)-ethyl]-6-methoxynaphthalene-2,3-dicarboxamide (**11**), 1-(3,4-dihydroxy-5-methoxyphenyl)-1,2-dihydroxy-7,8-dihydroxy-*N*-[(4-hydroxyphenyl)-ethyl]-*N*′-[(4-hydroxyphenyl) -ethyl]-6-methoxynaphthalene-2,3-dicarboxamide (**12**), 1-(3,4-dihydroxy-5-methoxyphenyl)-1,2-dihydroxy-7,8-dihydroxy-*N*,*N*′-bis-[2-(4-hydroxyphenyl)ethyl]-6-methoxynaphthalene-2,3-dicarboxamide (**13**), and 1-(3,4-dihydroxy-5-methoxyphenyl)-1,2-dihydroxy-6,7-dihydroxy-*N*,*N*′-bis-[2-(4-hydroxyphenyl)-ethyl]-8-methoxynaphthalene-2,3-dicarboxamide (**14**) [34], one new macrocyclic glycoside: 4-[(6′-*O*-*β*-D-allopyranosyl)-oxy]-hydroxy-benzoic acid cyclic dimeric inner ester (**5**) [35], one known phenolic amide: *N*-*trans*-caffeoyltyramine (**7**) [36], and two known C-glycosyl flavonoids: 2-[3-[2-*O-*(6-deoxy-α-L-mannopyranosyl)-*β*-D-glucopyranosyl]-4,5-dihydroxyphenyl]-5,7-dihydroxy-4H-1-benzopyran-4-one (**1**) and 2-[3-[2-*O*-(6-deoxy-*α*-L-mannopyranosyl)-*β*-D-glucopyranosyl]-4-hydroxyphenyl]-5,7-dihydroxy-4H-1-benzopyran-4-one (**3**) [37]. In addition, the detailed structural elucidation of five new compounds, including Compound **5** and Compounds **11**–**14**, was stated as below.

Compound **5**, UV (MeOH) *λ*_max_ (log *ε*) 201 (3.99), 248 (4.13). It was isolated as a light yellow amorphous solid powder. Its molecular formula was deduced to be C_26_H_28_O_14_, analyzed by UPLC-QTOF-MS/MS in the negative ion mode, with a [M + HCOOH − H]^−^ peak observed at *m/z* 609.1475 (calcd for C_26_H_28_O_14_, 609.1461), with fragment ions: 609, 563, 461, 419, 281, 239, 179, and 137. Combined with ^13^C NMR and the DEPT spectrum, the degree of unsaturation was supposed to be 13. Four doublet aromatic protons (*J* = 8.8 Hz) displayed in the ^1^H NMR spectrum could be presumed to be hydrogen signals on the *p*-substituted benzene ring, as shown in Table S1, Figures S3 and S9. The ^13^C NMR and DEPT spectrum (Table S1, Figures S4 and S10) showed 13 carbons ascribed to one methylene, nine methines (four aromatic methines), and three nonprotonated carbons (one carbonyl, one oxygenated aromatic carbon, and one aromatic carbon). Combined the degree of unsaturation with related literature, compound **5** could be speculated to be a dimer, which shared same skeleton with 4-[(6′-*O*-*β*–D-glucopyranosyl)-oxy]-hydroxy-benzoic acid cyclic dimeric inner ester [35]. Moreover, the ^13^C NMR spectrum also showed six carbon signals (*δ*_C_: 66.8, 70.0, 71.8, 72.9, 73.1, 98.4) corresponding to an allose sugar moiety [38]. The architecture of compound **5** was constructed mainly by 2D NMR data, as shown in Figure S2. Correlations (H-2/H-3, H-5/H-6, H-1′/H-2′, H-2′/H-3′, H-3′/H-4′, H-4′/H-5′, and H-5′/H-6′) observed in the ^1^H-^1^H COSY spectrum (Figures S2 and S5) suggested the presence of C-2-C-3, C-5-C-6, and C-1′-C-2′-C-3′-C-4′-C-5′-C-6′. The correlations of H-2/C-3, C-4, C-6, and C-7, and H-5/C-1, C-3, and C-4 in the HMBC spectrum (Figures S2 and S7) indicated the direct connection of benzene ring and carbonyl. Moreover, the correlation of H-1′/C-4 in the HMBC spectrum (Figures S2 and S7) allowed us to make sure the connection position of glycoside and the benzene ring. Correlations (H-3/H-1′, H-3/H-6′) observed in the NOESY spectrum (Figures S2 and S8) together with the coupling constant of the anomeric H-atoms (*J* = 8.0 Hz) indicated that the allose unit might belong to *β*-configuration [38]. Correlations (H-3/H-1′, H-3/H-6′ and H-6/H-6″′) observed in the NOESY spectrum (Figures S2 and S8) showed that compound **5** was a macrocyclic glycoside, named as 4-[(6′-*O*-*β*–D-allopyranosyl)-oxy]-hydroxy-benzoic acid cyclic dimeric inner ester after comparing with similar structures in previous reports [35,39,40,41].

Compound **14**, UV (MeOH) *λ*_max_ (log *ε*) 204 (4.71), 250 (4.27), 280 (3.98), 322 (4.04). It was isolated as a yellowish brown solid. Its molecular formula was deduced to be C_36_H_36_N_2_O_10_, analyzed by UPLC-QTOF-MS/MS in the negative ion mode, with a [M − H]^−^ peak observed at *m/z* 655.2293 (calcd for C_36_H_36_N_2_O_10_, 655.2297), with fragment ions: 655, 515, 352, 230, and 140. As shown in the ^1^H NMR (Table S3 and Figure S34), ^13^C-NMR (Table S3 and Figure S35), and 2D NMR (Figure S2) spectra, compound **14** possessed 14 quaternary carbons, 2 amide carbonyl groups, 2 aliphatic methines, 12 aromatic methines, 4 methylenes, and 2 methoxyls. These NMR data indicated that compound **14** showed a known skeleton of arylnaphthalene lignanamide [34]. The planar structure of compound **14** was analyzed according to the ^1^H-^1^H COSY and HMBC spectra. Correlations (H-*α*/H-*β*, H-*α*′/H-*β*′, H-2″/H-3″, H-2″′/H-3″′, H-5″/H-6″, H-5″′/H-6″′) observed in the ^1^H-^1^H COSY spectrum (Figures S2 and S36) suggested direct connection of C-α-C-β, C-α′-C-β′, C-2″ -C-3″, C-2″′-C-3″′, C-5″-C-6″, C-5″′-C-6″′. Moreover, the correlation of H-1/C-2a, H-2/C-2a, H-2/C-3a, and H-4/C-3a in the HMBC spectrum (Figures S2 and S38) allowed us to make sure the connection position of arylnaphthalene lignan and amide. Therefore, compound **14** was elucidated as 1-(3,4-dihydroxy-5-methoxyphenyl)-1,2-dihydroxy-6,7-dihydroxy-*N*,*N*′-bis-[2-(4-hydroxyphenyl)-ethyl]-8-methoxynaphthalene-2,3-dicarboxamide (**14**) [34]. New compounds **11**, **12**, and **13** shared the same arylnaphthalene lignanamide skeleton with compound **14** by comparison of their NMR and UPLC-QTOF-MS/MS data, and were identified by comparing the difference of the 2D NMR data (Figure S2).

Compound **11**, UV (MeOH) *λ*_max_ (log *ε*) 200 (4.70), 262 (4.19). It was isolated as a yellowish brown solid. Its molecular formula was deduced to be C_36_H_36_N_2_O_11_, analyzed by UPLC-QTOF-MS/MS in the negative ion mode, with a [M − H]^−^ peak observed at *m/z* 671.2241 (calcd for C_36_H_36_N_2_O_11_, 671.2246), with fragment ions: 671, 531, 368,336, 218, and 152. By contrast, the ^1^H-^1^H COSY spectrum (Figures S2 and S15) and HMBC spectrum (Figures S2 and S17) suggested that compound **11** bore hydroxy groups at the C-8′ and C-3″′ position, respectively. Thus, compound **11** was elucidated as 1-(3,4-dihydroxy-5-methoxyphenyl)-1,2-dihydroxy-7,8-dihydroxy-*N*-[(3,4-dihydroxyphenyl)-ethyl]-*N*′-[(4-hydroxyphenyl)-ethyl]-6-methoxynaphthalene-2,3-dicarboxamide [34].

Compound **12**, UV (MeOH) *λ*_max_ (log *ε*) 202 (4.16), 264 (3.85). It was isolated as a yellowish brown solid. Its molecular formula was deduced to be C_36_H_36_N_2_O_11_, analyzed by UPLC-QTOF-MS/MS in the negative ion mode, with a [M − H]^−^ peak observed at *m/z* 671.2239 (calcd for C_36_H_36_N_2_O_11_, 671.2246), with fragment ions: 671, 644, 531, 461, 352, 230, and 139. Similarly, compound **12** exhibited a hydroxy group at C-5″ according to the ^1^H-^1^H COSY spectrum (Figures S2 and S22) and HMBC spectrum (Figures S2 and S24) compared with compound **11**. Hence, compound **12** was elucidated as 1-(3,4-dihydroxy-5-methoxyphenyl)-1,2-dihydroxy-7,8-dihydroxy-*N*-[(4-hydroxyphenyl)-ethyl]-*N*′-[(4-hydroxyphenyl) -ethyl]-6-methoxynaphthalene-2,3-dicarboxamide [34].

Compound **13**, UV (MeOH) *λ*_max_ (log *ε*) 200 (4.79), 252 (4.30), 271 (4.14), 326 (3.99). It was isolated as a yellowish brown solid. Its molecular formula was deduced to be C_36_H_36_N_2_O_10_, analyzed by UPLC-QTOF-MS/MS in the negative ion mode, with a [M − H]^−^ peak observed at *m/z* 655.2287 (calcd for C_36_H_36_N_2_O_10_, 655.2297), with fragment ions: 655, 515, 501, 352, 230, 174, 139. As mentioned above, compound **13** was an isomer of compound **14**, which had a methoxy group at C-6 position rather than C-8 position, elucidated as 1-(3,4-dihydroxy-5-methoxyphenyl)-1,2-dihydroxy-7,8-dihydroxy-*N*,*N*′-bis-[2-(4-hydroxyphenyl)ethyl]-6-methoxynaphthalene-2,3-dicarboxamide [34].

### 3.4. Antioxidant Activities of Compounds Isolated from WUE-A4

**Figure 6:**
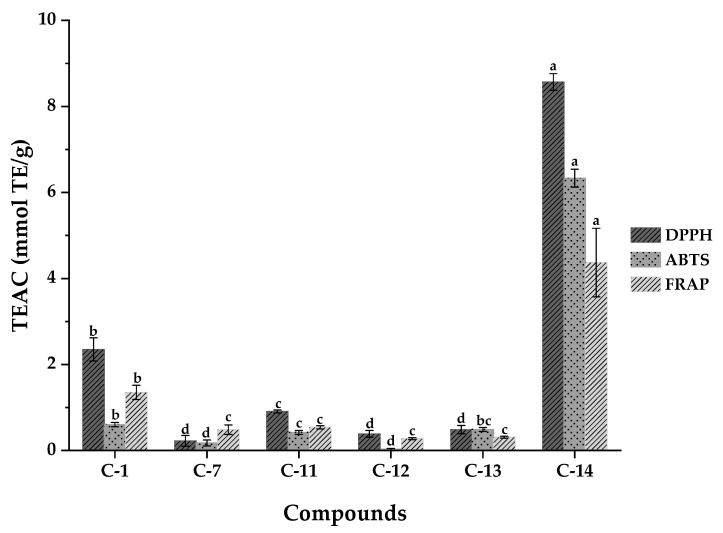
Trolox equivalent antioxidant capacity (TEAC) of compounds isolated from WUE-A4 tested by DPPH, ABTS and FRAP assays. Means labeled by different letters (^a–d^) were significantly different at a level of *p* < 0.05 (n = 3) by DMRT (Duncan’s multiple range test).

Antioxidant activities of WUE-A4 and compounds isolated in WUE-A4 were evaluated with DPPH, ABTS, and FRAP assays to further explore their potential total antioxidant capacities. As shown in Table 4 and Figure 6, compound **14** exhibited the highest DPPH and ABTS radical scavenging activities with the IC_50_ values of 6.405 ± 0.362 µM and 5.381 ± 0.092 µM, which were lower than the IC_50_ values of Trolox (35.973 ± 1.102 µM and 22.353 ± 0.568 µM). Moreover, compound **14** also showed the highest ferric reducing antioxidant capacities with a FRAP value of 17.488 ± 1.625 mmol Fe^2+^/g, which was higher than the FRAP value of Trolox (16.212 ± 1.271 mmol Fe^2+^/g). Moreover, compound **1** also displayed excellent antioxidant activities. It is a C-glycosyl flavonoid that has been found in the leaves of *Cinnamosma fragrans* by Nomoto et al. [37], though there has been no other antioxidant report about it since. In summary, it could be speculated that the remarkable antioxidant activities of compounds **1** and **14** might be highly correlated with their strong binding affinity capacities to SOD and XOD. Moreover, the results showed that the antioxidant activities of other compounds were lower than compounds **1** and **14**, judging from the results of the preliminary antioxidant activities screening.

### 3.5. Anti-inflammatory Activities of Compounds Isolated from WUE-A4

**Figure 7:**
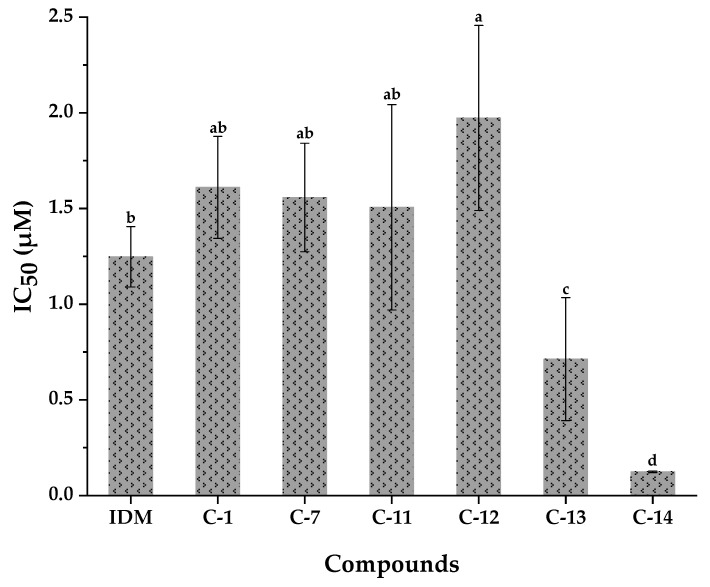
COX-2 inhibitory activities of compounds isolated from WUE-A4. IDM, Indomethacin; Means labeled by different letters (^a–d^) were significantly different at a level of *p* < 0.05 (n = 3) by DMRT (Duncan’s multiple range test).

Previous studies have shown that free oxygen radicals participate in the pathogenesis of many diseases by damaging important biological macromolecules such as proteins and DNA, and then cause pathological reactions such as cancer and inflammation [42]. Accordingly, inflammation is one of the most important symptoms imposed by oxidative stress. COX-2 is a key enzyme which catalyzes the conversion of arachidonic acid (AA) to prostaglandin G_2_ (PGG_2_), and then catalyzes a sequential enzyme reaction to transform PGG_2_ into prostaglandin H_2_ (PGH_2_). Meanwhile, COX-2 can also be induced to overexpress when macrophages, fibroblasts, endothelial cells, and monocytes undergo inflammation, which are closely associated with inflammation and cancer [43]. As shown in Figure 7, compound **14** also exhibited the highest inhibitory activities against COX-2 with an IC_50_ value of 0.123 ± 0.004 µM, which was lower than that of indomethacin (1.248 ± 0.158 µM). Meanwhile, compound **13** also displayed relatively higher inhibitory capacities on COX-2 with an IC_50_ value of 0.713 ± 0.322 µM, which was lower than the positive control of indomethacin. In addition, compounds **1**, **7**, and **11** exerted almost the same inhibitory activities on COX-2 compared with the positive control of indomethacin. In this regard, many studies reported the anti-inflammatory activities of compound **7**; for example, it showed good inhibitory effects against LPS-induced NO production in RAW 264.7 macrophages [44,45,46].

## 4. Conclusions

To date, as a traditional medicinal plant in local communities in Africa, the potential bioactive components with noteworthy antioxidant and anti-inflammatory activity in *W. ugandensis* and its correlated mechanisms have not been explored. To meet and solve this challenge, three different antioxidant assays, including DPPH, ABTS, and FRAP, were firstly used to trace the antioxidant activities of crude extracts and fractions from stem barks of *W. ugandensis*. Then, the bio-affinity ultrafiltration combining SOD and XOD with LC-MS/MS was used to rapidly screen out 9 and 12 bioactive components against SOD and XOD from the antioxidant effective fraction WUE-A4, respectively. As a result, eight compounds, including four new lignanamides, one new macrocyclic glycoside, and three known compounds, were successfully isolated and identified from WUE-A4, which greatly improved the phytochemical knowledge on the bioactive constituents from *W. ugandensis*. Then, the antioxidant activities revealed that compounds **14** and **1** showed higher antioxidant activities than the positive control of Trolox, suggesting they would be the potential natural antioxidants from *W. ugandensis*. In addition, the potential antioxidant activities of compounds **14** and **1** might be highly related to their strong binding affinities with SOD and XOD. More strikingly, compounds **14**, **13**, **1**, **7**, and **11** expressed noteworthy inhibitory activities on COX-2, comparable to or even better than that of indomethacin, a positive drug control, which is in common clinical use. Especially, the new compound **14** also displayed the best anti-inflammatory activity, much better than that of clinical indomethacin. In summary, this study showcased an alternative and integrative strategy to quickly screen for and to subsequently identify the most potential natural antioxidants combing multiple target enzymes with affinity ultra-filtration LC-MS from the crude extracts of a medicinal plant of interest, and would provide a good guidance to search for other natural antioxidants from other natural products. Moreover, it was also revealed for the first time that new lignanamides could be the bioactive components of *W. ugandensis* with the potentials to exert prominent antioxidant and anti-inflammatory activities, which is of great interest for further exploration in the near future. On the other hand, besides the individual potentials of the singular chemical moieties that were isolated and discussed, the bouquet of the compounds identified together in their entirety may work synergistically to unfold the discussed biological effects of *W. ugandensis*.

## Figures and Tables

**Table 1:** Antioxidant activities of different extracts from stem barks of *W. ugandensis* tested by DPPH, ABTS, and FRAP assays.

Sample	DPPH ^#^	ABTS ^#^	FRAP ^#^
	IC_50_ (µg/mL)	IC_50_ (µg/mL)	mmol Fe^2+^/g
Trolox	9.0 ± 0.3 ^f^	5.9 ± 0.1 ^e^	16.2 ± 1.3 ^a^
WUZ	18.9 ± 0.3 ^d^	10.2 ± 0.5 ^d^	1.5 ± 0.1 ^c^
WUP	219.1 ± 1.4 ^a^	85.5 ± 8.2 ^a^	0.3 ± 0.1 ^f^
WUE	17.8 ± 0.3 ^e^	9.4 ± 0.5 ^d,e^	5.6 ± 0.3 ^b^
WUN	46.5 ± 0.5 ^b^	22.5 ± 3.9 ^c^	1.1 ± 0.1 ^d^
WUW	33.8 ± 0.6 ^c^	32.8 ± 1.6 ^b^	0.6 ± 0.1 ^e^

^#^ Data were expressed as means ± standard deviation (n = 3). WUZ, 95% EtOH crude extract; WUP, petroleum ether fraction; WUE, ethyl acetate fraction, WUN, n-butanol fraction; WUH, H_2_O fraction; DPPH, 1,1-diphenyl-2-picrylhydrazyl; ABTS, 2,2′-azinobis-(3-ethylbenzthiazoline-6-sulfonic acid); FRAP, ferric-reducing antioxidant power; IC_50_, concentration acquired when DPPH and ABTS radicals were 50% inhibited; FRAP value was represented as mmol Fe^2+^/g of sample; Means labeled by different letters ^(a–f)^ were significantly different at a level of *p* < 0.05 (n = 3) by DMRT (Duncan’s multiple range test); -, Not tested.

**Table 2:** Antioxidant activities of different fractions eluted from WUE tested by DPPH, ABTS, and FRAP assays.

Sample	DPPH ^#^	ABTS ^#^	FRAP ^#^
	IC_50_ (µg/mL)	IC_50_ (µg/mL)	mmol Fe^2+^/g
WUE-A	18.4 ± 0.3 ^g^	10.4 ± 0.5 ^f^	3.4 ± 0.3 ^b^
WUE-B	16.4 ± 0.3 ^g^	9.6 ± 0.5 ^f^	7.6 ± 0.4 ^a^
WUE-C	24.7 ± 0.4 ^f^	10.0 ± 0.8 ^f^	2.9 ± 0.7 ^b^
WUE-D	55.6 ± 0.9 ^d^	19.0 ± 3.0 ^d^	1.0 ± 0.2 ^c^
WUE-E	35.5 ± 0.5 ^e^	14.8 ± 1.2 ^e^	0.6 ± 0.1 ^cd^
WUE-F	62.3 ± 0.8 ^c^	25.4 ± 0.9 ^c^	0.6 ± 0.1 ^c,d^
WUE-G	243.5 ± 4.2 ^a^	82.2 ± 3.8 ^a^	0.4 ± 0.0 ^d^
WUE-H	139.9 ± 1.4 ^b^	52.3 ± 4.3 ^b^	0.4 ± 0.0 ^c,d^

^#^ Data were expressed as means ± standard deviation (n = 3). WUE, ethyl acetate fraction; WUE-A–WUE-H, eight fractions eluted from WUE. DPPH, 1,1-diphenyl-2-picrylhydrazyl; ABTS, 2,2′-azinobis-(3-ethylbenzthiazoline-6-sulfonic acid); FRAP, ferric-reducing antioxidant power; IC_50_, concentration acquired when DPPH and ABTS radicals were 50% inhibited; FRAP value was represented as mmol Fe^2+^/g of sample; Means labeled by different letters (^a–g^) were significantly different at a level of *p* < 0.05 (n = 3) by DMRT (Duncan’s multiple range test).

**Table 3:** The identification and RBAs of potential ligands against xanthine oxidase and superoxide dismutase in WUE-A4.

No.	LC-MS/MS					UF-RBA ^#^	
	Rt (min)	*m/z*	MS/MS-Fragments	Compounds	SMILES *	XOD	SOD
**1**	11.8	593	593, 446, 428, 393, 369, 353, 338, 326, 310, 297, 284, 230, 187	2-[3-[2-*O*-(6-deoxy-*α*-L-mannopyranosyl)-*β*-D-glucopyranosyl]-4,5-dihydroxyphenyl]-5,7-dihydroxy-4*H*-1-benzopyran-4-one	O=C1C2=C(O)C=C(O)C=C2OC(C3=CC(O)=C(O)C([C@@H]4[C@@H](O[C@@H]5[C@@H](O)[C@@H](O)[C@H](O)[C@@H](C)O5)[C@H](O)[C@@H](O)[C@H](CO)O4)=C3)=C1	1.2 ± 0.6 ^c,d^	1.9 ± 0.1 ^a,b,c^
**2**	14.3	609	609, 352, 301, 284, 271, 254, 216, 192, 162	Isomer of 5	-	1.8 ± 0.1 ^b,c^	-
**3**	17.5	577	577, 430, 413, 395, 364, 352, 322, 310, 292, 281, 268, 212, 158, 59	2-[3-[2-*O*-(6-deoxy-*α*-L-mannopyranosyl)-*β*-D-glucopyranosyl]-4-hydroxyphenyl]-5,7-dihydroxy-4*H*-1-benzopyran-4-one	O=C1C2=C(O)C=C(O)C=C2OC(C3=CC=C(O)C([C@@H]4[C@@H](O[C@@H]5[C@@H](O)[C@@H](O)[C@H](O)[C@@H](C)O5)[C@H](O)[C@@H](O)[C@H](CO)O4)=C3)=C1	1.0 ± 0.2 ^d^	2.0 ± 0.1 ^a,b^
**4**	20.6	625	625, 579, 433, 311, 121, 112	Unknown	-	0.6 ± 0.1 ^d^	1.6 ± 0.3 ^b,c,d,e^
**5**	22.7	609	609, 563, 500, 461, 391, 361, 328, 298, 137, 108, 90, 62	4-[(6′-*O*-*β*-D-allopyranosyl)-oxy]-hydroxy-benzoic acid cyclic dimeric inner ester *	O[C@@H]1[C@@H](COC(C2=CC=C(O3)C=C2)=O)O[C@@H](OC4=CC=C(C(OC[C@H]5O[C@@H]3[C@H](O)[C@H](O)[C@@H]5O)=O)C=C4)[C@H](O)[C@@H]1O	1.2 ± 0.1 ^c,d^	1.1 ± 0.1 ^e^
**6**	25.0	617	617, 205, 186, 163, 131, 114, 101	Unknown	-	2.1 ± 0.3 ^b^	1.5 ± 0.4 ^d,e^
**7**	27.5	298	297, 255, 227, 190, 147, 135, 107	*N*-trans-caffeoyltyramine	O=C(NCCC1=CC=C(O)C=C1)/C=C/C2=CC(O)=C(O)C=C2	1.1 ± 0.3 ^c,d^	1.1 ± 0.3 ^e^
**8**	28.5	327	327, 312, 206, 163, 150, 134	Unknown	-	2.4 ± 0.2 ^b^	1.9 ± 0.5 ^b,c,d^
**9**	29.9	671	671, 530, 491, 475, 453, 418, 392, 367, 352, 338, 299, 282, 229	Isomer of 11 and 12	-	-	-
**10**	30.5	471	471, 403, 373, 289, 263, 235, 208, 150	Unknown	-	2.3 ± 0.4 ^b^	-
**11**	32.1	671	671, 597, 580, 555, 531, 516, 491, 352, 337, 245, 230, 179	1-(3,4-dihydroxy-5-methoxyphenyl)-1,2-dihydroxy-7,8-dihydroxy-*N*-[(3,4-dihydroxyphenyl)ethyl]-*N*′-[(4-hydroxyphenyl)ethyl]-6-methoxynaphthalene-2,3-dicarboxamide *	OC1=C(OC)C=C2C(C(C3=CC(O)=C(O)C(OC)=C3)C(C(N([H])CCC4=CC=C(O)C=C4)=O)C(C(N([H])CCC5=CC=C(O)C(O)=C5)=O)=C2)=C1O	-	-
**12**	33.1	671	671, 588, 531, 516, 490, 368, 352, 337, 260, 231, 178	1-(3,4-dihydroxy-5-methoxyphenyl)-1,2-dihydroxy-7,8-dihydroxy-*N*-[(4-hydroxyphenyl)ethyl]-*N*′-[(4-hydroxyphenyl)ethyl]-6-methoxynaphthalene-2,3-dicarboxamide *	OC1=C(OC)C=C2C(C(C3=CC(O)=C(O)C(OC)=C3)C(C(N([H])CCC4=CC=C(O)C(O)=C4)=O)C(C(N([H])CCC5=CC=C(O)C=C5)=O)=C2)=C1O	1.7 ± 0.5 ^b,c^	-
**13**	36.1	655	655, 514, 491, 477, 392, 364, 336, 312, 175	1-(3,4-dihydroxy-5-methoxyphenyl)-1,2-dihydroxy-7,8-dihydroxy-*N*,*N*′-bis-[2-(4-hydroxyphenyl)ethyl]-6-methoxynaphthalene-2,3-dicarboxamide *	OC1=C(OC)C=C2C(C(C3=CC(O)=C(O)C(OC)=C3)C(C(N([H])CCC4=CC=C(O)C=C4)=O)C(C(N([H])CCC5=CC=C(O)C=C5)=O)=C2)=C1O	1.3 ± 0.2 ^c,d^	1.3 ± 0.1 ^e^
**14**	38.5	655	655, 514, 500, 476, 440, 402, 392, 363, 351, 336	1-(3,4-dihydroxy-5-methoxyphenyl)-1,2-dihydroxy-6,7-dihydroxy-*N*,*N*′-bis-[2-(4-hydroxyphenyl)ethyl]-8-methoxynaphthalene-2,3-dicarboxamide *	OC1=C(O)C(OC)=C(C(C2=CC(O)=C(O)C(OC)=C2)C(C(N([H])CCC3=CC=C(O)C=C3)=O)C(C(N([H])CCC4=CC=C(O)C=C4)=O)=C5)C5=C1	3.8 ± 0.5 ^a^	1.5 ± 0.1 ^c,d,e^

^#^ Data were expressed as means ± standard deviation (n = 3). * SMILES were acquired by Chemoffice 18.0. Rt, retention time; UF, ultrafiltration; RBA, relative binding affinity; XOD, xanthine oxidase; SOD, superoxide dismutase; Means labeled by different letters (^a–e^) were significantly different at a level of *p* < 0.05 (n = 3) by DMRT (Duncan’s multiple range test); *, new compounds; -, No binding affinity.

**Table 4:** Antioxidant activities of compounds isolated from WUE-A4 tested by DPPH, ABTS, and FRAP assays.

Compounds	DPPH ^#^	ABTS ^#^	FRAP ^#^
	IC_50_ (µM)	IC_50_ (µM)	mmol Fe^2+^/g
**Trolox**	36.0 ± 1.1 ^b^	22.4 ± 0.6 ^c^	16.2 ± 1.3 ^a^
**WUE-A4**	10.5 ± 0.7 ^b,^*	9.2 ± 1.2 ^c,^*	7.1 ± 0.6 ^b^
**1**	25.8 ± 1.4 ^b^	62.5 ± 4.2 ^c^	5.4 ± 0.7 ^b^
**7**	635.8 ± 289.5 ^a^	462.7 ± 157.3 ^b^	1.9 ± 0.1 ^c^
**11**	59.1 ± 4.1 ^b^	80.0 ± 10.3 ^c^	2.2 ± 0.1 ^c^
**12**	142.5 ± 36.1 ^b^	1151.8 ± 629.7 ^a^	1.1 ± 0.2 ^c^
**13**	114.9 ± 20.8 ^b^	69.8 ± 6.3 ^c^	1.3 ± 0.2 ^c^
**14**	6.4 ± 0.4 ^b^	5.4 ± 0.1^c^	17.5 ± 1.6 ^a^

^#^ Data were expressed as means ± standard deviation (n = 3). * The unit of data was µg/mL. DPPH, 1,1-diphenyl-2-picrylhydrazyl; ABTS, 2,2′-azinobis-(3-ethylbenzthiazoline-6-sulfonic acid); FRAP, ferric-reducing antioxidant power; IC_50_, Concentration when DPPH radicals were 50% inhibited; FRAP value was represented as mmol Fe^2+^/g of sample; Means labeled by different letters (^a–c^) were significantly different at a level of *p* < 0.05 (n = 3) by DMRT (Duncan’s multiple range test).

## Data Availability

We confirm the copyright of figures and tables in our manuscript, which are our original data, and not from other publications.

## Supplementary Materials

The following are available online at https://www.mdpi.com/2076-3921/10/3/370/s1, Figure S1: Flow diagram of extractions, fractions and compounds from *W. ugandensis*, Figure S2: Selected ^1^H-^1^H COSY, HMBC and NOESY correlations for new compounds, Figure S3: ^1^H NMR (600 MHz) spectrum of compound **5** in Methanol-*d*_4_, Figure S4: ^13^C NMR and DEPT (150 MHz) spectrum of compound **5** in Methanol-*d*_4_, Figure S5: ^1^H-^1^H COSY (600 MHz) spectrum of compound **5** in Methanol-*d*_4_, Figure S6: HSQC (600 MHz) spectrum of compound **5** in Methanol-*d*_4_, Figure S7: HMBC (600 MHz) spectrum of compound **5** in Methanol-*d*_4_, Figure S8: NOESY (600 MHz) spectrum of compound **5** in Methanol-*d*_4_, Figure S9: ^1^H NMR (600 MHz) spectrum of compound **5** in DMSO-*d*_6_, Figure S10: ^13^C NMR and DEPT (150 MHz) spectrum of compound **5** in DMSO-*d*_6_, Figure S11: UPLC-QTOF-MS spectrum of compound **5**, Figure S12: UPLC-QTOF-MS/MS spectrum of compound **5**, Figure S13: ^1^H NMR (600 MHz) spectrum of compound **11** in Methanol-*d*_4_, Figure S14: ^13^C NMR and DEPT (150 MHz) spectrum of compound **11** in Methanol-*d*_4_, Figure S15: ^1^H-^1^H COSY (600 MHz) spectrum of compound **11** in Methanol-*d*_4_, Figure S16: HSQC (600 MHz) spectrum of compound **11** in Methanol-*d*_4_, Figure S17: HMBC (600 MHz) spectrum of compound **11** in Methanol-*d*_4_, Figure S18: UPLC-QTOF-MS spectrum of compound **11**, Figure S19: UPLC-QTOF-MS/MS spectrum of compound **11**, Figure S20: ^1^H NMR (600 MHz) spectrum of compound **12** in Methanol-*d*_4_, Figure S21: ^13^C NMR and DEPT (150 MHz) spectrum of compound **12** in Methanol-*d*_4_, Figure S22: ^1^H-^1^H COSY (600 MHz) spectrum of compound **12** in Methanol-*d*_4_, Figure S23: HSQC (600 MHz) spectrum of compound **12** in Methanol-*d*_4_, Figure S24: HMBC (600 MHz) spectrum of compound **12** in Methanol-*d*_4_, Figure S25: UPLC-QTOF-MS spectrum of compound **12**, Figure S26: UPLC-QTOF-MS/MS spectrum of compound **12**, Figure S27. ^1^H NMR (600 MHz) spectrum of compound **13** in Methanol-*d*_4_, Figure S28: ^13^C NMR and DEPT (150 MHz) spectrum of compound **13** in Methanol-*d*_4_, Figure S29: ^1^H-^1^H COSY (600 MHz) spectrum of compound **13** in Methanol-*d*_4_, Figure S30: HSQC (600 MHz) spectrum of compound **13** in Methanol-*d*_4_, Figure S31: HMBC (600 MHz) spectrum of compound **13** in Methanol-*d*_4_, Figure S32: UPLC-QTOF-MS spectrum of compound **13**, Figure S33: UPLC-QTOF-MS/MS spectrum of compound **13**, Figure S34: ^1^H NMR (600 MHz) spectrum of compound **14** in Methanol-*d*_4_, Figure S35: ^13^C NMR and DEPT (150 MHz) spectrum of compound **14** in Methanol-*d*_4_, Figure S36: ^1^H-^1^H COSY (600 MHz) spectrum of compound **14** in Methanol-*d*_4_, Figure S37: HSQC (600 MHz) spectrum of compound **14** in Methanol-*d*_4_, Figure S38: HMBC (600 MHz) spectrum of compound **14** in Methanol-*d*_4_, Figure S39: UPLC-QTOF-MS spectrum of compound **14**, Figure S40: UPLC-QTOF-MS/MS spectrum of compound **14**, Table S1: ^1^H and ^13^C NMR data of compound **5** (Methanol-*d*_4_ and DMSO-*d*_6_), Table S2: ^1^H and ^13^C NMR data of compound **11** and **12** (Methanol-*d*_4_), Table S3: ^1^H and ^13^C NMR data of compound **13** and **14** (Methanol-*d*_4_).
