# Supplementary material for: New Lignanamides with Antioxidant and Anti-Inflammatory Activities Screened Out and Identified from Warburgia ugandensis Combining Affinity Ultrafiltration LC-MS with SOD and XOD Enzymes
Source: Antioxidants (Basel). 2021 Mar 1;10(3):370. doi: 10.3390/antiox10030370 (PMC8001783; doi:10.3390/antiox10030370)
Supplement: Supplementary file 1 [file antioxidants-10-00370-s001.pdf]

antioxidants

MDPI

Supporting Information

New Lignanamides with Antioxidant and Anti-inflammatory Activities Screened out and Identified from *Warburgia ugandensis* Combining Affinity Ultrafiltration LC-MS with SOD and XOD Enzymes

Xiao-Cui Zhuang [1,2,3,4,5], Gui-Lin Chen [1,3,4], Ye Liu [1,3,4], Yong-Li Zhang [1,3,4] and Ming-Quan Guo [1,3,4*]

1 Key Laboratory of Plant Germplasm Enhancement and Specialty Agriculture, Wuhan Botanical Garden, Chinese Academy of Sciences, Wuhan 430074, China; zhuangxiaocui@yxnu.edu.cn (X. C. Z.); glchen@wbgcas.cn (G. L. C.); liuye@wbgcas.cn (Y. L); zhangyongli@wbgcas.cn (Y. L. Z.)
2 University of Chinese Academy of Sciences, Beijing 100049, China;
3 Sino-Africa Joint Research Center, Chinese Academy of Sciences, Wuhan 430074, China;
4 Innovation Academy for Drug Discovery and Development, Chinese Academy of Sciences, Shanghai 201203, China;
5 School of Chemical Biology and Environment, Yuxi Normal University, Yuxi 653100, China;
* Correspondence: guomq@wbgcas.cn; Tel.: +86-027-87700850

Contents of Supporting Information

Figure S1. Flow diagram of extractions, fractions and compounds from *W. ugandensis*............4

Table S1. ^{1}H and ^{13}C NMR data of compound **5** (Methanol-d_4 and DMSO-d_6)...........................5

Table S2. ^{1}H and ^{13}C NMR data of compound **11** and **12** (Methanol-d_4)......................................6

Table S3. ^{1}H and ^{13}C NMR data of compound **13** and **14** (Methanol-d_4)......................................7

Figure S2. Selected ^{1}H-^{1}H COSY, HMBC and NOESY correlations for new compounds...........8

Figure S3. ^{1}H NMR (600 MHz) spectrum of compound **5** in Methanol-d_4 9

Figure S4. ^{13}C NMR and DEPT (150 MHz) spectrum of compound **5** in Methanol-d_4................9

Figure S5. ^{1}H-^{1}H COSY (600 MHz) spectrum of compound **5** in Methanol-d_4...........................10

Figure S6. HSQC (600 MHz) spectrum of compound **5** in Methanol-d_4 10

Figure S7. HMBC (600 MHz) spectrum of compound **5** in Methanol-d_4 11

Figure S8. NOESY (600 MHz) spectrum of compound **5** in Methanol-d_4...................................11

Figure S9. ^{1}H NMR (600 MHz) spectrum of compound **5** in DMSO-d_6 12

Figure S10. ^{13}C NMR and DEPT (150 MHz) spectrum of compound **5** in DMSO-d_6.................12

Figure S11. UPLC-QTOF-MS spectrum of compound **5**...13

Figure S12. UPLC-QTOF-MS/MS spectrum of compound **5**..13

Figure S13. ^{1}H NMR (600 MHz) spectrum of compound **11** in Methanol-d_4 14

Figure S14. ^{13}C NMR and DEPT (150 MHz) spectrum of compound **11** in Methanol-d_4..........14

Figure S15. ^{1}H-^{1}H COSY (600 MHz) spectrum of compound **11** in Methanol-d_4.......................15

Figure S16. HSQC (600 MHz) spectrum of compound **11** in Methanol-d_4 15

Figure S17. HMBC (600 MHz) spectrum of compound **11** in Methanol-d_4 16

Figure S18. UPLC-QTOF-MS spectrum of compound **11**..16

Figure S19. UPLC-QTOF-MS/MS spectrum of compound **11**...17

Figure S20. ^{1}H NMR (600 MHz) spectrum of compound **12** in Methanol-d_4 17

Figure S21. ^{13}C NMR and DEPT (150 MHz) spectrum of compound **12** in Methanol-d_4..........18

Figure S22. ^{1}H-^{1}H COSY (600 MHz) spectrum of compound **12** in Methanol-d_4.......................18

Figure S23. HSQC (600 MHz) spectrum of compound **12** in Methanol-d_4 19

Figure S24. HMBC (600 MHz) spectrum of compound **12** in Methanol-d_4 19

Figure S25. UPLC-QTOF-MS spectrum of compound **12**..20

Figure S26. UPLC-QTOF-MS/MS spectrum of compound **12**...20

Figure S27. ^{1}H NMR (600 MHz) spectrum of compound **13** in Methanol-*d*$_4$ 21

Figure S28. ^{13}C NMR and DEPT (150 MHz) spectrum of compound **13** in Methanol-*d*$_4$...........21

Figure S29. ^{1}H-^{1}H COSY (600 MHz) spectrum of compound **13** in Methanol-*d*$_4$.......................22

Figure S30. HSQC (600 MHz) spectrum of compound **13** in Methanol-*d*$_4$ 22

Figure S31. HMBC (600 MHz) spectrum of compound **13** in Methanol-*d*$_4$ 23

Figure S32. UPLC-QTOF-MS spectrum of compound **13**..23

Figure S33. UPLC-QTOF-MS/MS spectrum of compound **13**...24

Figure S34. ^{1}H NMR (600 MHz) spectrum of compound **14** in Methanol-*d*$_4$ 24

Figure S35. ^{13}C NMR and DEPT (150 MHz) spectrum of compound **14** in Methanol-*d*$_4$...........25

Figure S36. ^{1}H-^{1}H COSY (600 MHz) spectrum of compound **14** in Methanol-*d*$_4$.......................25

Figure S37. HSQC (600 MHz) spectrum of compound **14** in Methanol-*d*$_4$ 26

Figure S38. HMBC (600 MHz) spectrum of compound **14** in Methanol-*d*$_4$ 26

Figure S39. UPLC-QTOF-MS spectrum of compound **14**..27

Figure S40. UPLC-QTOF-MS/MS spectrum of compound **14**...27

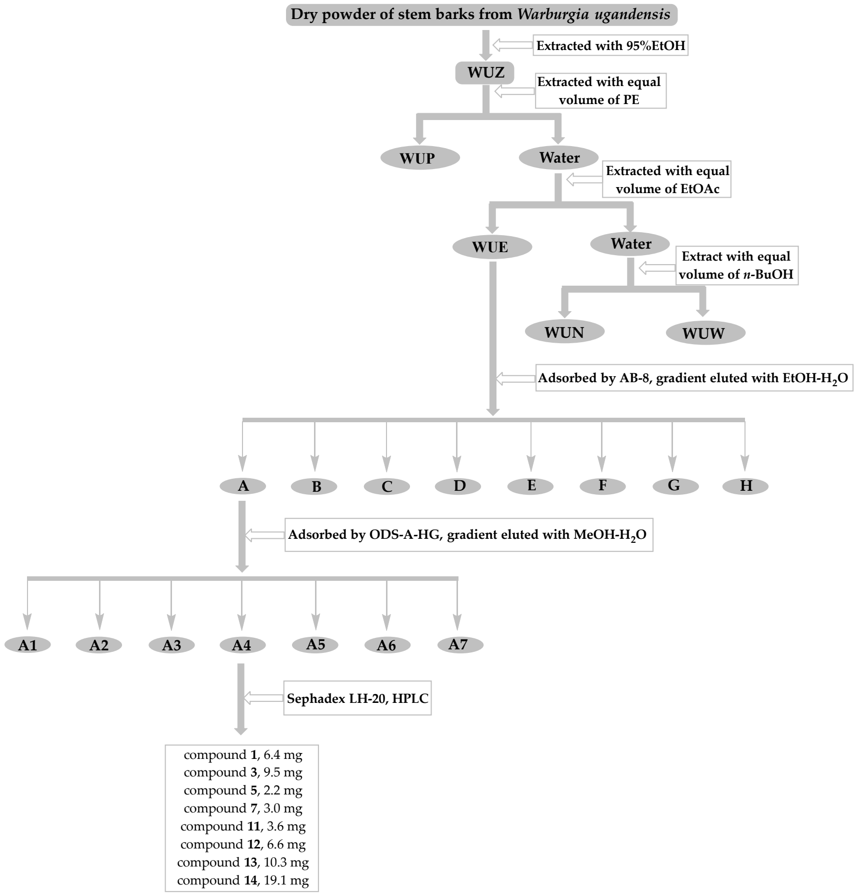

Figure S1. Flow diagram of extractions, fractions and compounds from *W. ugandensis*. WUZ, 95% EtOH crude extract; WUP, petroleum ether fraction; WUE, ethyl acetate fraction; WUN, *n*-butanol fraction; WUH, H_2O fraction; PE, petroleum ether; EtOAc: ethyl acetate; *n*-BuOH, *n*-butyl alcohol; EtOH, ethanol; MeOH, methanol; HPLC, high performance liquid chromatography.

Table S1. ^{1}H and ^{13}C NMR data of compound **5** (Methanol-d_4 and DMSO-d_6)

Position	Methanol-$d_4$$^\#$		DMSO-$d_6$$^\#$	
	δ_H	δ_C	δ_H	δ_C
1,1′′		124.5 (s)		122.5 (s)
2, 6, 2′′, 6′′	8.11 (2H, d, *J* = 8.8 Hz)	132.6 (d)	8.02 (2H, d, *J* = 8.9 Hz)	131.3 (d)
3, 5, 3′′, 5′′	7.27 (2H, d, *J* = 8.8 Hz)	117.1 (d)	7.19 (2H, d, *J* = 8.9 Hz)	115.8 (d)
4, 4′′		162.6 (s)		160.6 (s)
7, 7′′		167.4 (s)		165.1 (s)
1′, 1′′′	5.53 (1H, d, *J* = 8.0 Hz)	98.4 (d)	5.39 (1H, d, *J* = 8.0 Hz)	96.8 (d)
2′, 2′′′	3.75 (1H, dd, *J* = 8.0, 3.0 Hz)	71.8 (d)	3.55 (1H, dd, *J* = 8.0, 3.0 Hz)	71.5 (d)
3′, 3′′′	4.23 (1H, overlap)	73.1 (d)	4.00 (1H, d, *J* = 3.0 Hz)	71.2 (d)
4′, 4′′′	3.61 (1H, dd, *J* = 10.0, 3.0 Hz)	70.0 (d)	3.45 (1H, d, *J* = 10.2 Hz)	69.9 (d)
5′, 5′′′	4.41 (1H, td, *J* = 10.0, 1.8 Hz)	72.9 (d)	4.27 (1H, td, *J* = 10.2, 2.1 Hz)	68.3 (d)
6′, 6′′′	4.24 (1H, overlap)<br>4.55 (1H, dd, *J* = 10.0, 1.8 Hz)	66.8 (t)	4.10 (1H, t, *J* = 11.0 Hz)<br>4.37 (1H, dd, *J* = 11.0, 2.1 Hz)	65.4 (t)

$^\#$: 600 MHz for ^{1}H NMR and 150 MHz for ^{13}C NMR.

Table S2. ^{1}H and ^{13}C NMR data of compound **11** and **12** (Methanol-d_4)

**No.**	Compound **11**		No.	Compound **12**	
	δ_H	δ_C		δ_H	δ_C
1	4.79 (1H, s)	41.1, d	1	4.78 (1H, s)	41.0, d
2	3.71 (1H, s)	50.4, d	2	3.769 (1H, s)	50.4, d
2a		174.3, s	2a		174.2, s
3		135.4, s	3		135.3, s
3a		170.1, s	3a		170.0, s
4	7.26 (1H, s)	135.8, d	4	7.27 (1H, s)	135.8, d
4a		124.3, s	4a		124.3, s
5	6.55 (1H, s)	105.3, d	5	6.55 (1H, s)	105.4, d
6		148.3, s	6		148.2, s
6-OMe	3.90 (3H, s)	56.6, q	6-OMe	3.90 (3H, s)	56.6, q
7		137.7, s	7		137.7, s
8		144.7, s	8		144.7, s
8a		118.5, s	8a		118.5, s
1′		126.6, s	1′		126.6, s
2′	6.37 (1H, d, J = 1.9 Hz)	104.1, d	2′	6.37 (1H, d, J = 1.9 Hz)	104.1, d
3′		149.3, s	3′		149.3, s
3′-OMe	3.74 (3H, s)	56.5, q	3′-OMe	3.74 (3H, s)	56.5, q
4′		133.5, s	4′		133.5, s
5′		146.2, s	5′		146.2, s
6′	6.06 (1H, d, J = 1.9 Hz)	109.2, d	6′	6.06 (1H, d, J = 1.9 Hz)	109.2, d
α	3.20 (2H, dt, J = 13.4, 6.9 Hz)	42.5, t	α	3.23(2H, t, J = 6.9 Hz)	42.2, t
β	2.52 (2H, td, J = 6.9, 2.8 Hz)	35.4, t	β	2.47 (2H, td, J = 6.9, 3.2 Hz)	35.5, t
α'	3.36 (2H, t, J =7.4 Hz)	42.7, t	α'	3.37 (2H, Dt, J = 11.2, 7.3 Hz)	42.8, t
β'	2.63 (2H, t, J = 7.4 Hz)	35.9, t	β'	2.69 (2H, t, J = 7.3 Hz)	35.6, t
1′′		131.1, s	1′′		131.7, s
2′′, 6′′	6.82 (2H, d, J = 7.9 Hz)	130.7, d	2′′	6.53 (1H, d, J = 2.0 Hz)	116.7, d
3′′, 5′′	6.65 (2H, overlap)	116.2, d	3′′		145.9, s
4′′		156.7, s	4′′		144.6, s

1‴		132.1, s	5″	6.62 (1H, d, *J* = 7.9 Hz)	116.3, d
2‴	6.64 (1H, overlap)	116.9, d	6″	6.46 (1H, dd, *J* = 7.9, 2.0 Hz)	121.1, d
3‴		145.9, s	1‴		131.4, s
4‴		144.6, s	2‴, 6‴	6.96 (2H, d, *J* = 7.9 Hz)	130.8, d
5‴	6.65 (1H, overlap)	116.3, d	3‴, 5‴	6.67 (2H, d, *J* = 7.9 Hz)	116.2, d
6‴	6.46 (1H, dd, *J* = 7.9, 2.0 Hz)	121.1, d	4‴		156.8, s

Table S3. ^{1}H and ^{13}C NMR data of compound **13** and **14** (Methanol-d_4)

**No.**	Compound **13**		No.	Compound **14**	
	δ_H	δ_C		δ_H	δ_C
1	4.80 (1H, s)	41.0, d	1	4.79 (1H, s)	41.4, d
2	3.70 (1H, s)	50.4, d	2	3.68 (1H, s)	50.4, d
2a		174.3, s	2a		174.0, s
3		135.4, s	3		135.5, s
3a		170.0, s	3a		170.0, s
4	7.27 (1H, s)	135.8, d	4	7.23 (1H, s)	135.2, d
4a		124.3, s	4a		124.6, s
5	6.54 (1H, s)	105.3, d	5	6.68 (1H, s)	112.9, d
6		148.3, s	6		146.5, s
6-OMe	3.89 (3H, s)	56.6, q	7		142.0, s
7		137.7, s	8		147.4, s
8		144.6, s	8-OMe	3.54 (3H, s)	60.9, q
8a		118.5, s	8a		123.5, s
1′		126.6, s	1′		126.8, s
2′	6.38 (1H, d, *J* = 1.9 Hz)	104.1, d	2′	6.34 (1H, d, *J* = 2.0 Hz)	104.0, d
3′		149.3, s	3′		149.4, s
3′-OMe	3.73 (3H, s)	56.5, q	3′-OMe	3.73 (3H, s)	56.5, q
4′		133.4, s	4′		133.5, s
5′		145.9, s	5′		146.0, s
6′	6.06 (1H, d, *J* = 1.9 Hz)	109.2, d	6′	6.06 (1H, d, *J* = 2.0 Hz)	109.3, d
α	3.19 (2H, dt, *J* = 13.4, 6.8 Hz)	42.4, t	*α*	3.16 (2H, dt, *J* = 13.8, 6.8 Hz)	42.5, t
β	2.52 (2H, td, *J* = 6.8, 3.7 Hz)	35.4, t	*β*	2.51 (2H, td, *J* = 6.8, 3.7 Hz)	35.5, t
α′	3.37 (2H, hept, *J* = 6.8 Hz)	42.7, t	*α*′	3.34 (2H, hept, *J* = 6.8 Hz)	42.7, t
β′	2.68 (2H, t, *J* = 6.8 Hz)	35.6, t	*β*′	2.66 (2H, t, *J* = 6.8 Hz)	35.6, t
1″		131.1, s	1″		131.2, s
2″, 6″	6.81 (2H, d, *J* = 7.9 Hz)	130.7, d	2″, 6″	6.81 (2H, d, *J* = 8.1 Hz)	130.7, d
3″, 5″	6.64 (2H, d, *J* = 7.9 Hz)	116.2, d	3″, 5″	6.65(2H, d, *J* = 8.1 Hz)	116.2, d

4′′		156.7, s	4′′		156.7, s
1′′′		131.4, s	1′′′		131.3, s
2′′′, 6′′′	6.95 (2H, d, *J* = 7.9 Hz)	130.8, d	2′′′, 6′′′	6.94 (2H, d, *J* = 8.1 Hz)	130.8, d
3′′′, 5′′′	6.67 (2H, d, *J* = 7.9 Hz)	116.3, d	3′′′, 5′′′	6.65 (2H, d, *J* = 8.1 Hz)	116.3, d
4′′′		156.8, s	4′′′		156.8, s

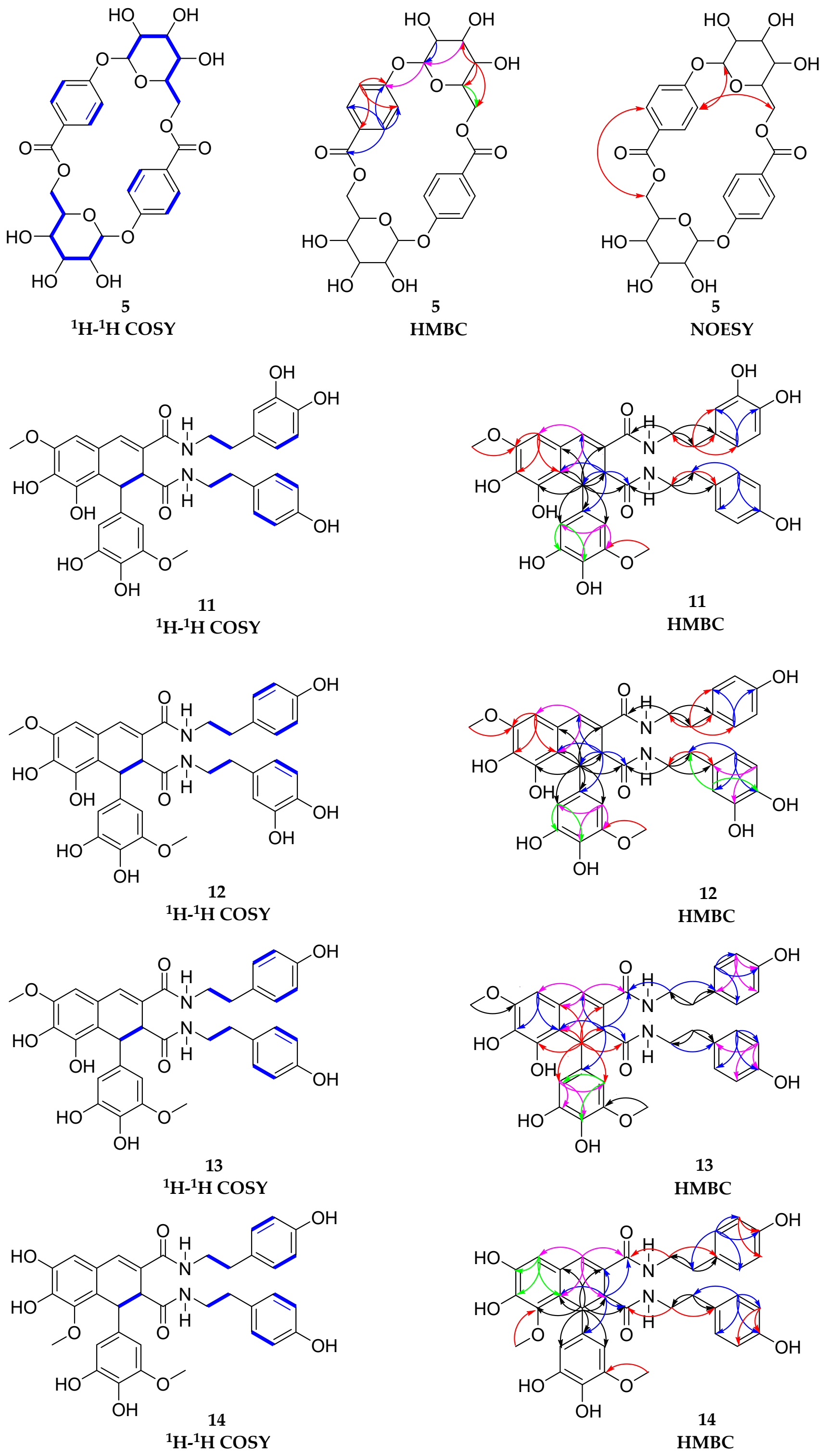

Figure S2. Selected ^{1}H-^{1}H COSY, HMBC and NOESY correlations for compound **5**, **11**, **12**, **13** and **14**.

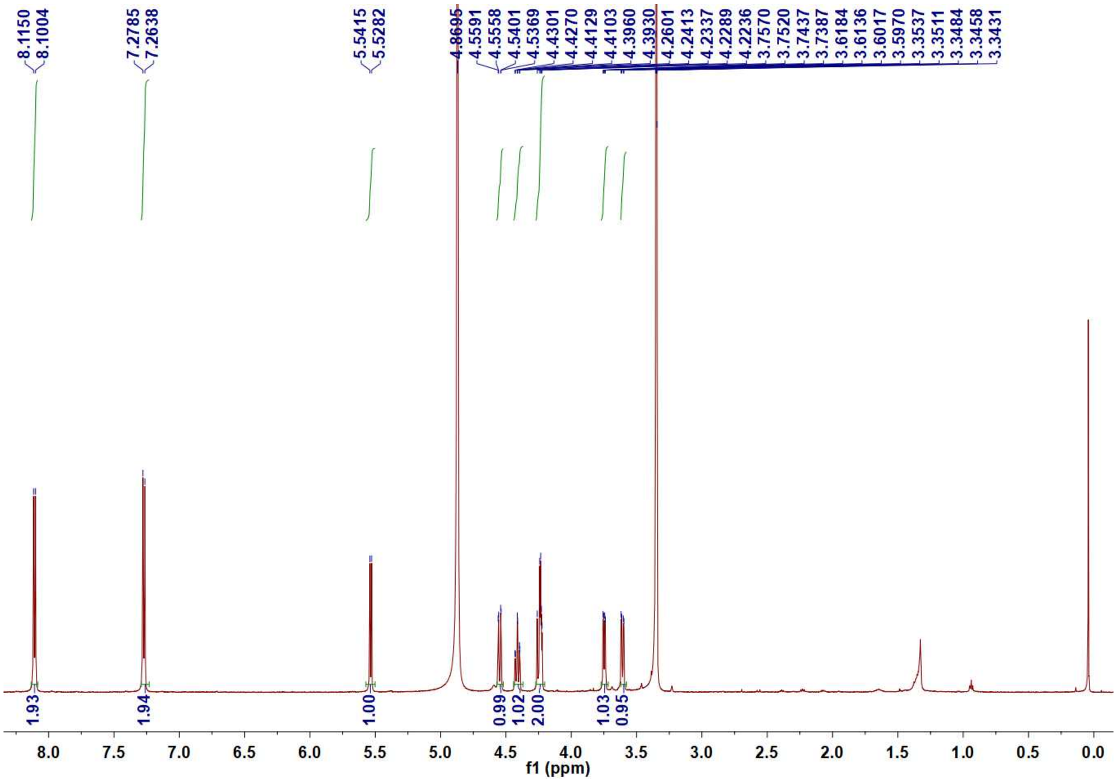

Figure S3. ^{1}H NMR (600 MHz) spectrum of compound **5** in Methanol-d_4

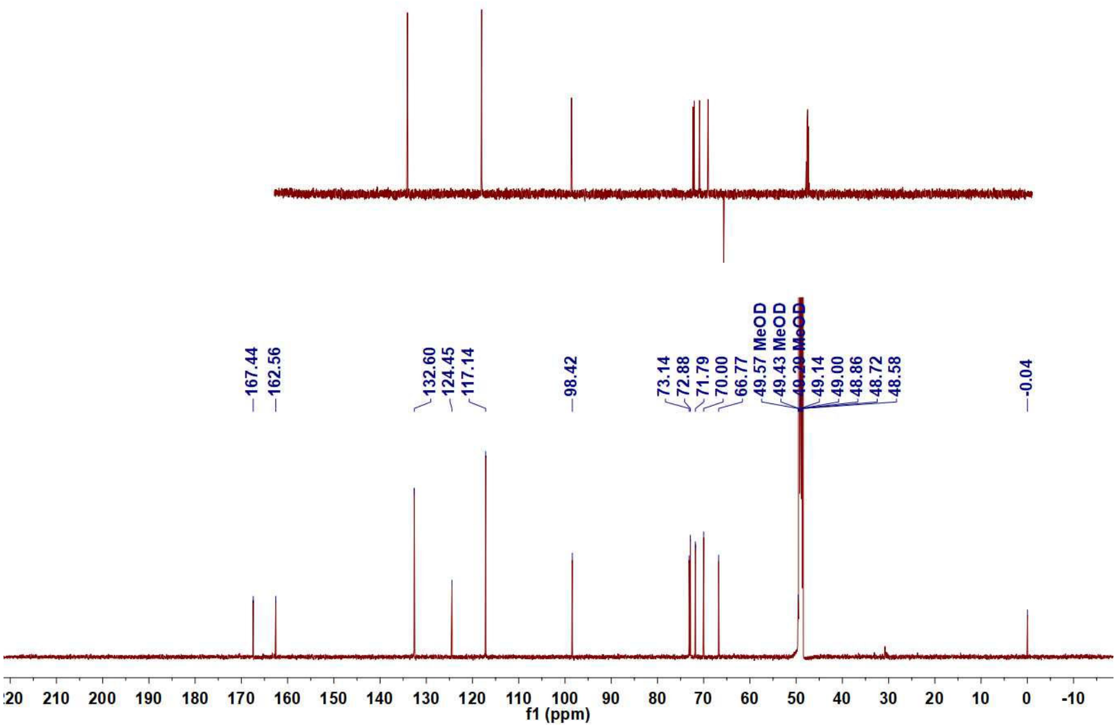

Figure S4. ^{13}C NMR and DEPT (150 MHz) spectrum of compound **5** in Methanol-d_4

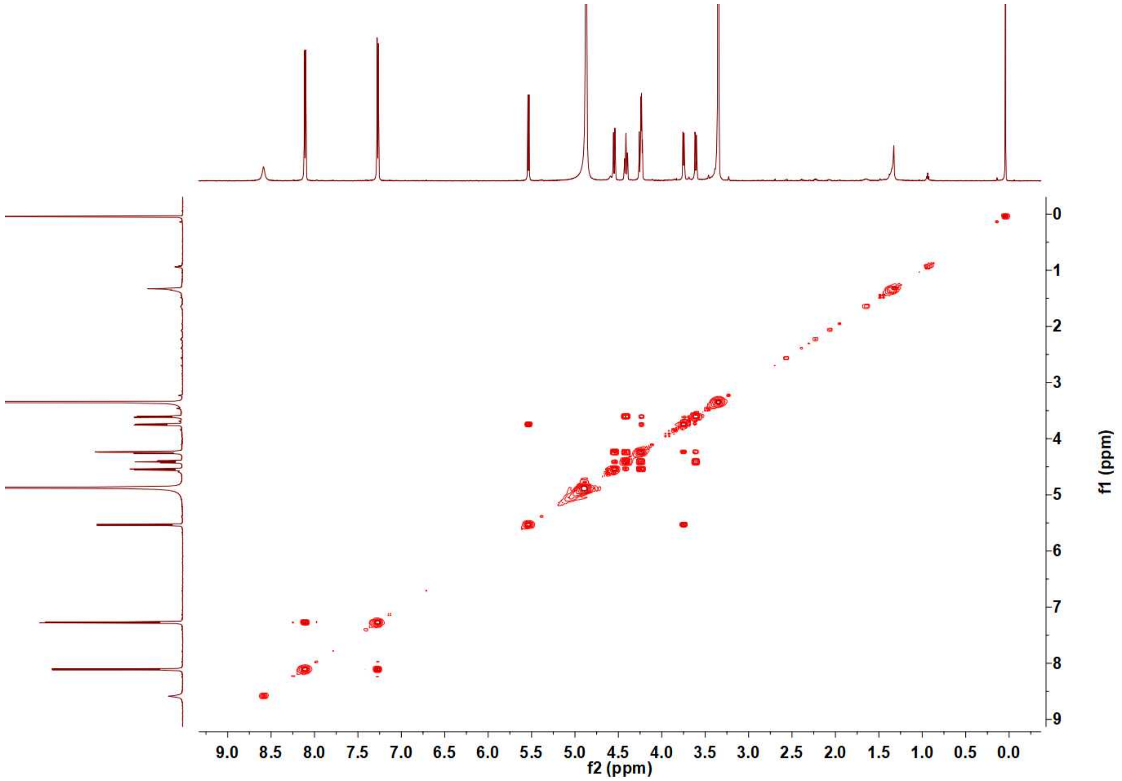

Figure S5. ^{1}H-^{1}H COSY (600 MHz) spectrum of compound **5** in Methanol-d_4

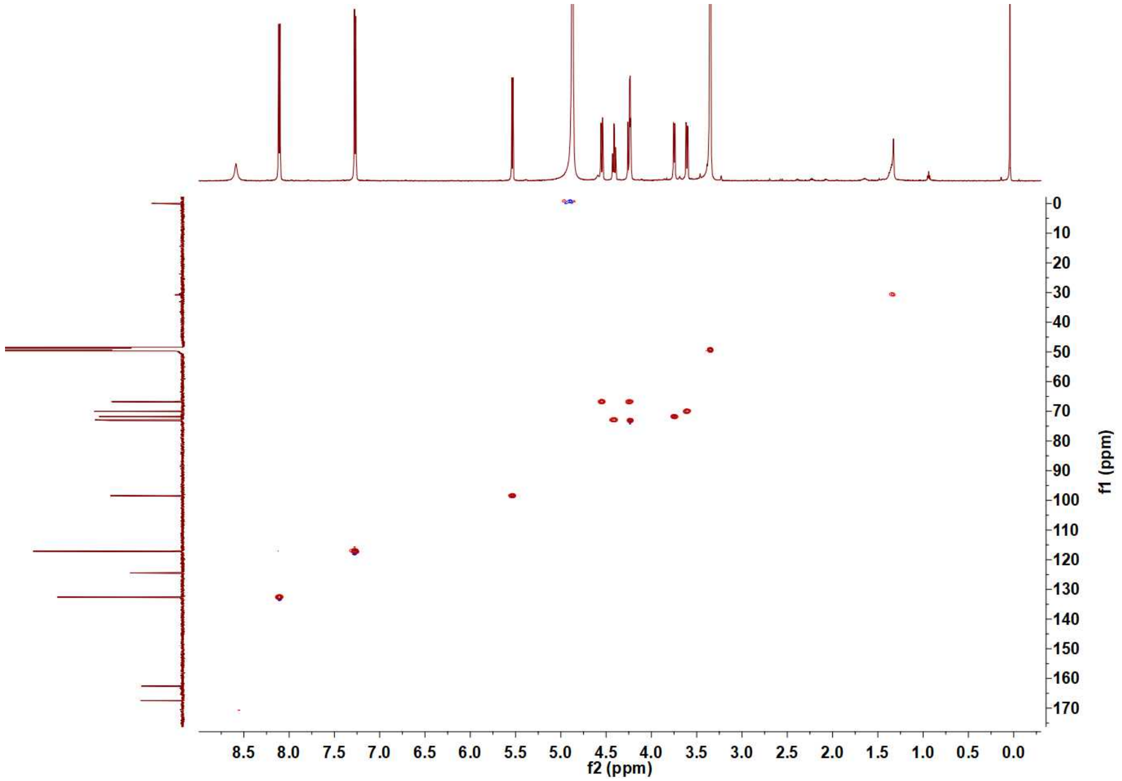

Figure S6. HSQC (600 MHz)spectrum of compound **5** in Methanol-d_4

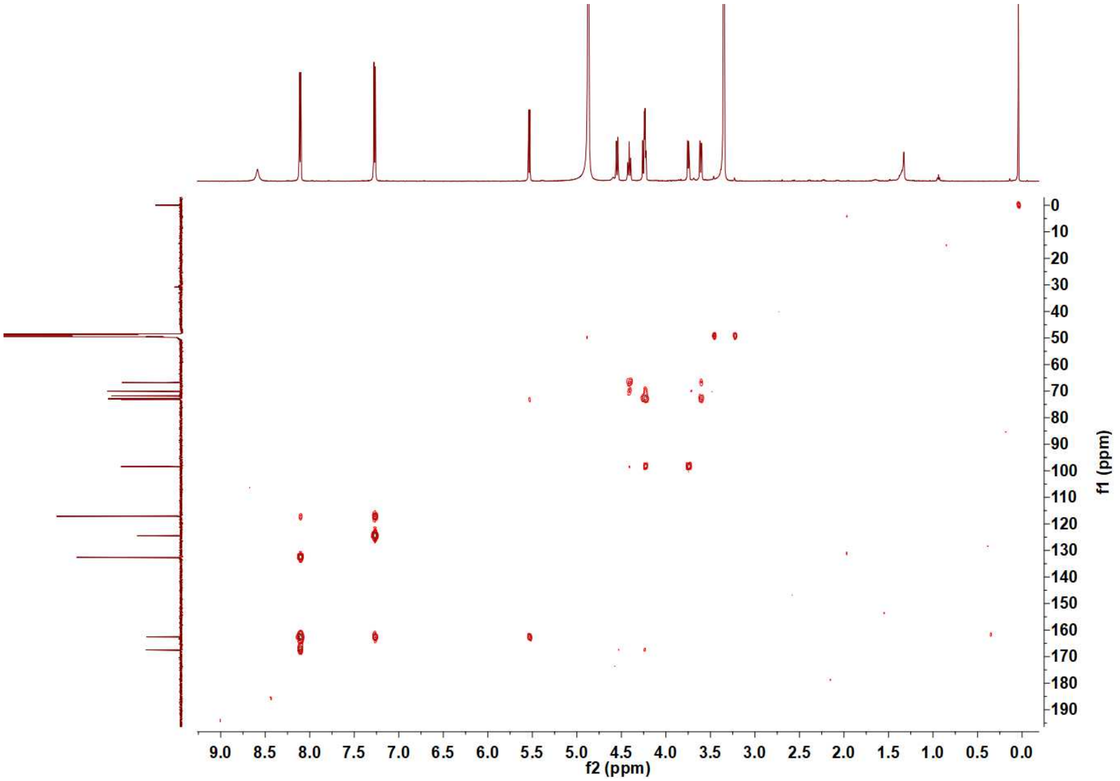

Figure S7. HMBC (600 MHz) spectrum of compound **8** in Methanol-d_4

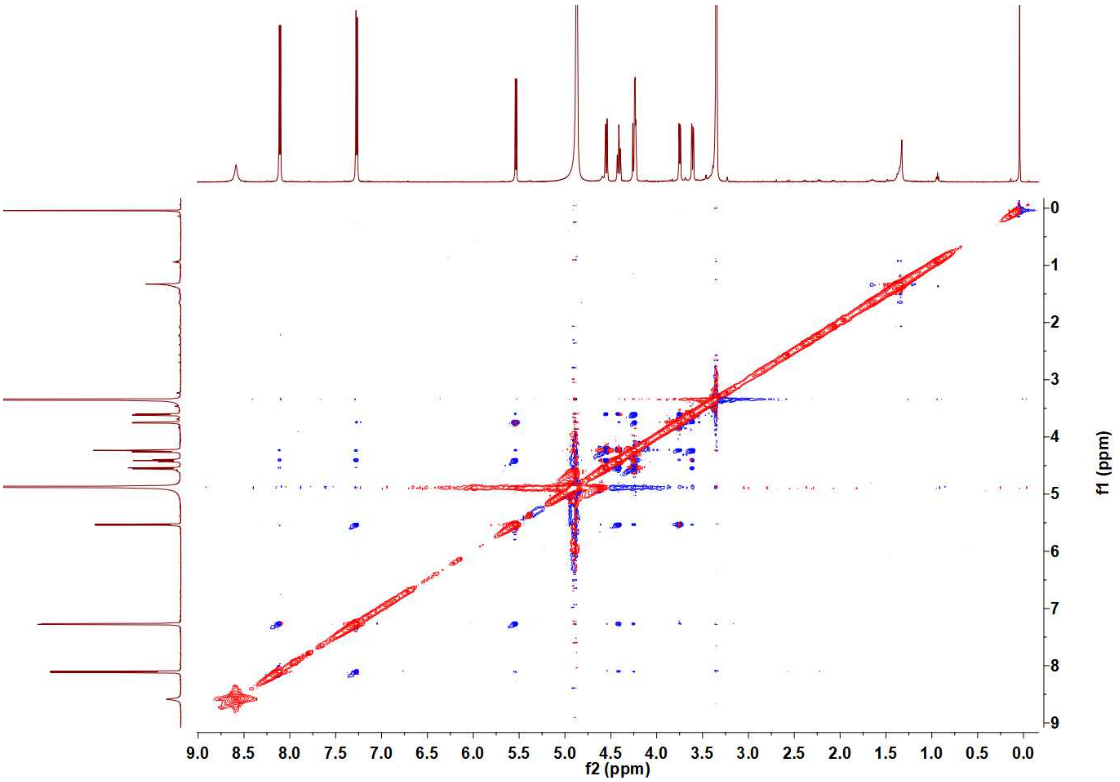

Figure S8. NOESY (600 MHz) spectrum of compound **5** in Methanol-d_4

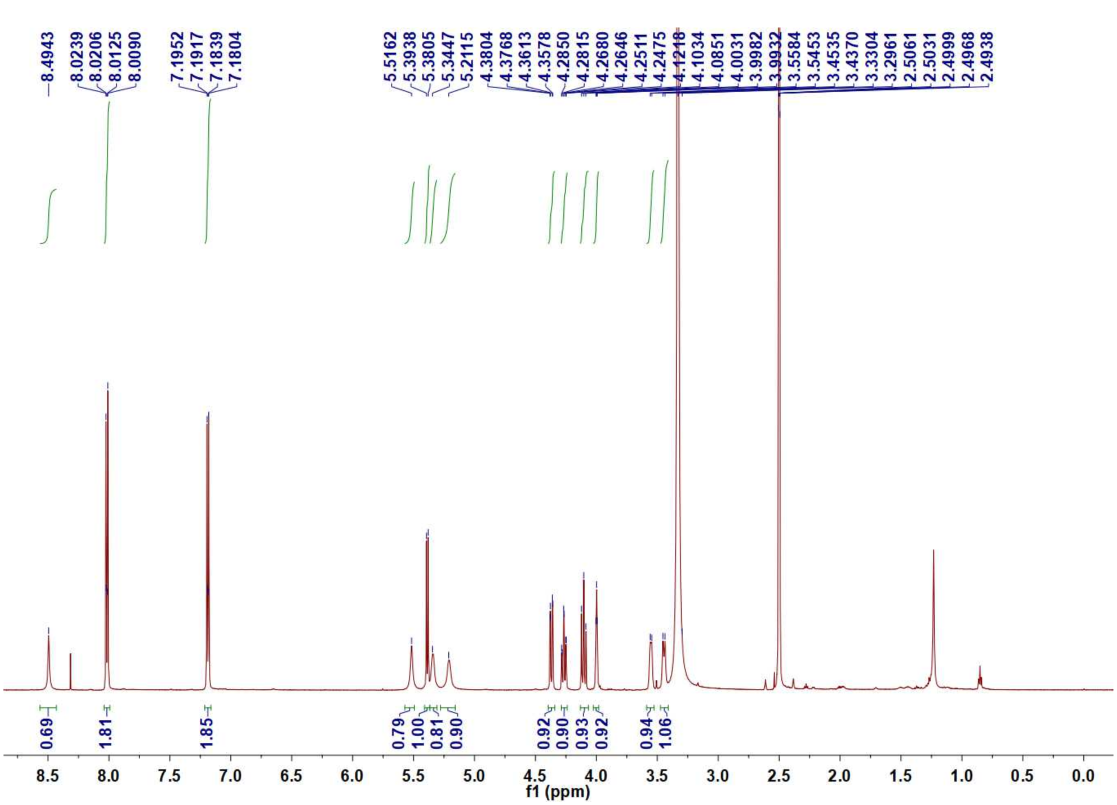

Figure S9. ^{1}H NMR (600 MHz) spectrum of compound **5** in DMSO-d_6

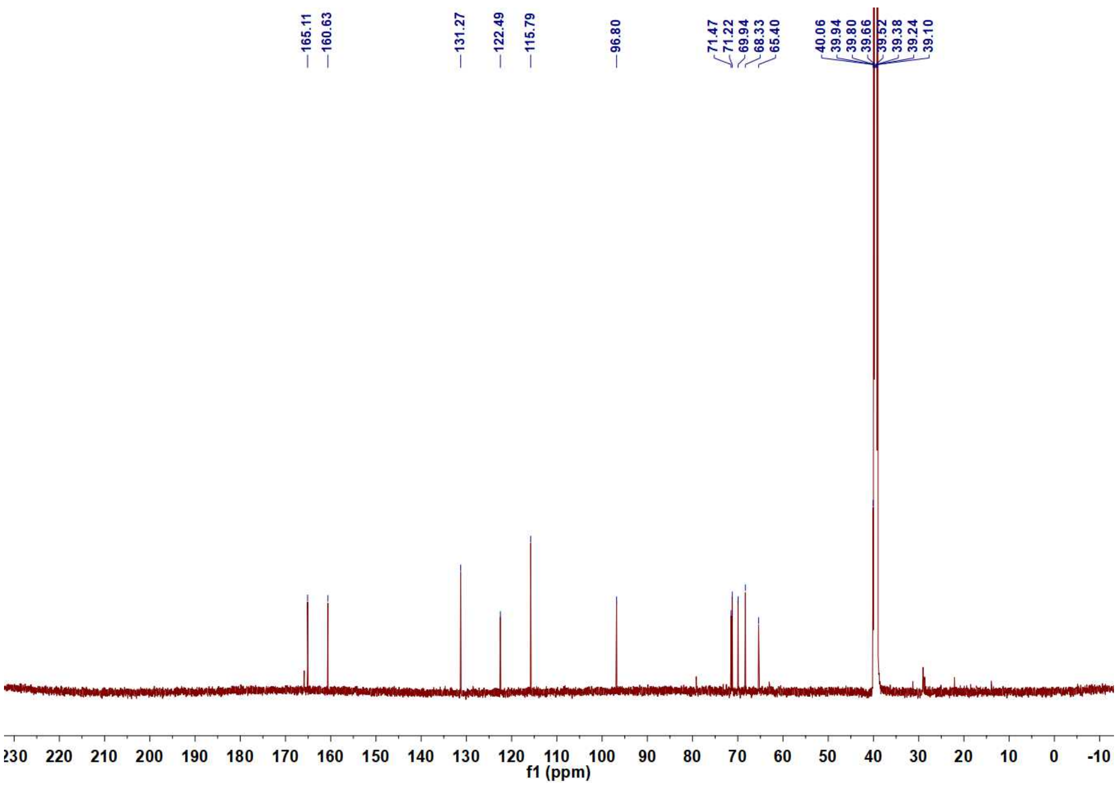

Figure S10. ^{13}C NMR and DEPT (150 MHz) spectrum of compound **5** in DMSO-d_6

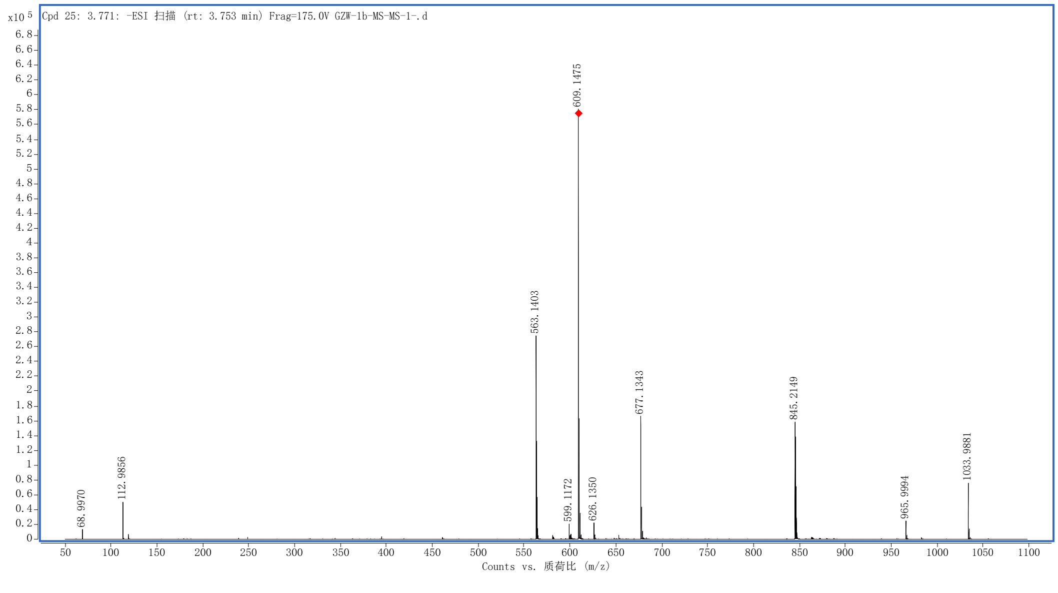

Figure S11. UPLC-QTOF-MS spectrum of compound **5**

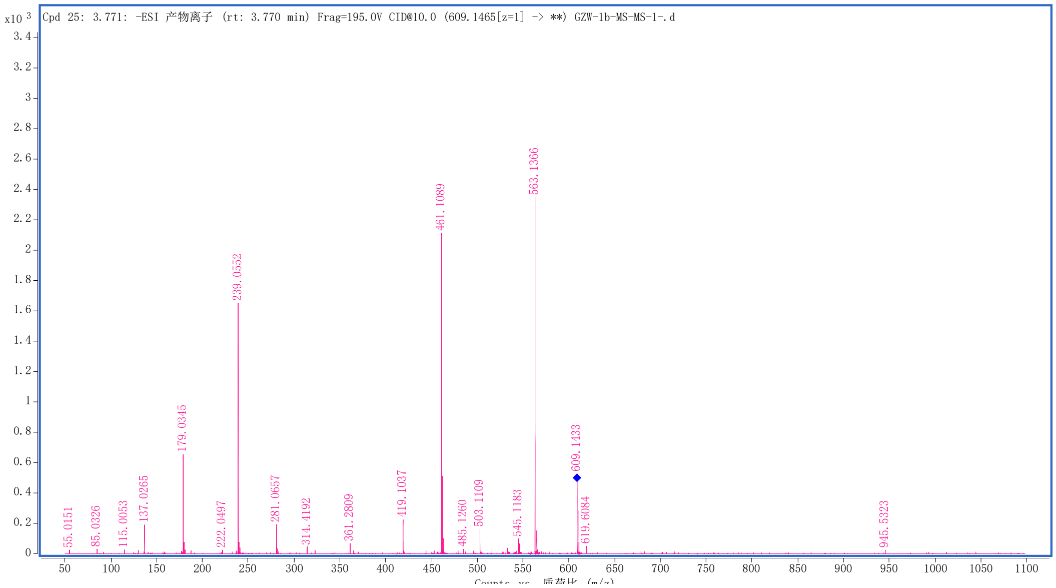

Figure S12. UPLC-QTOF-MS/MS spectrum of compound **5**

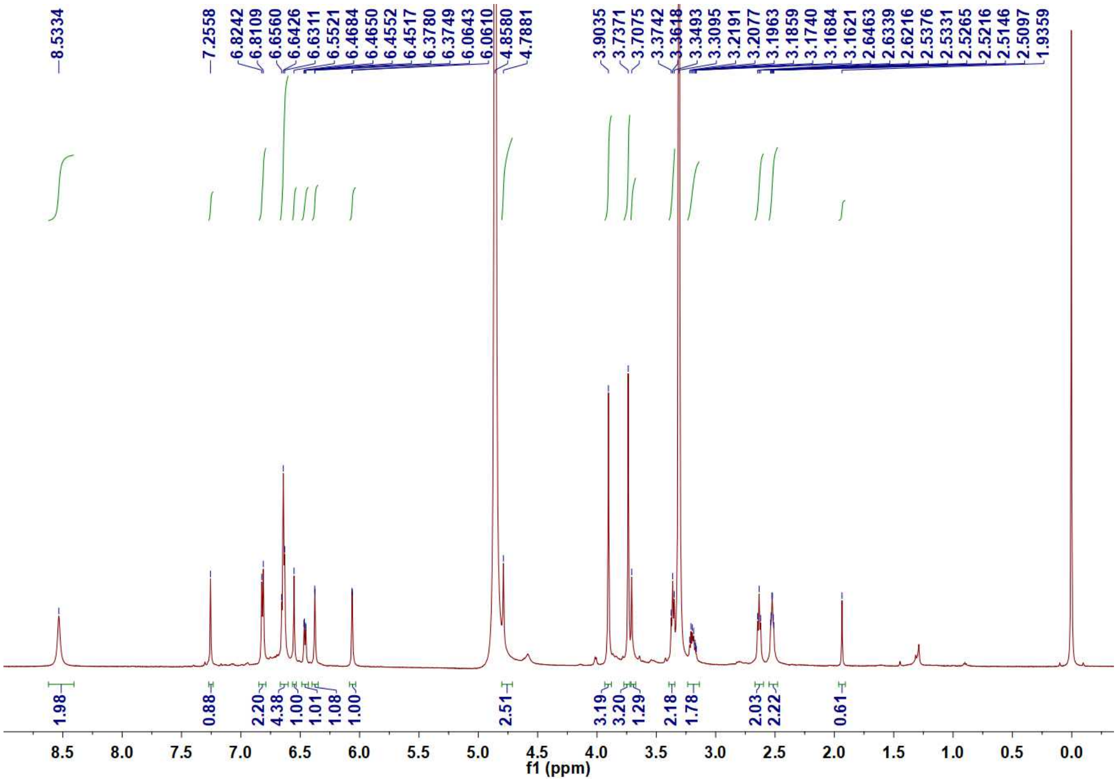

Figure S13. ^{1}H NMR (600 MHz) spectrum of compound **11** in Methanol-d_4

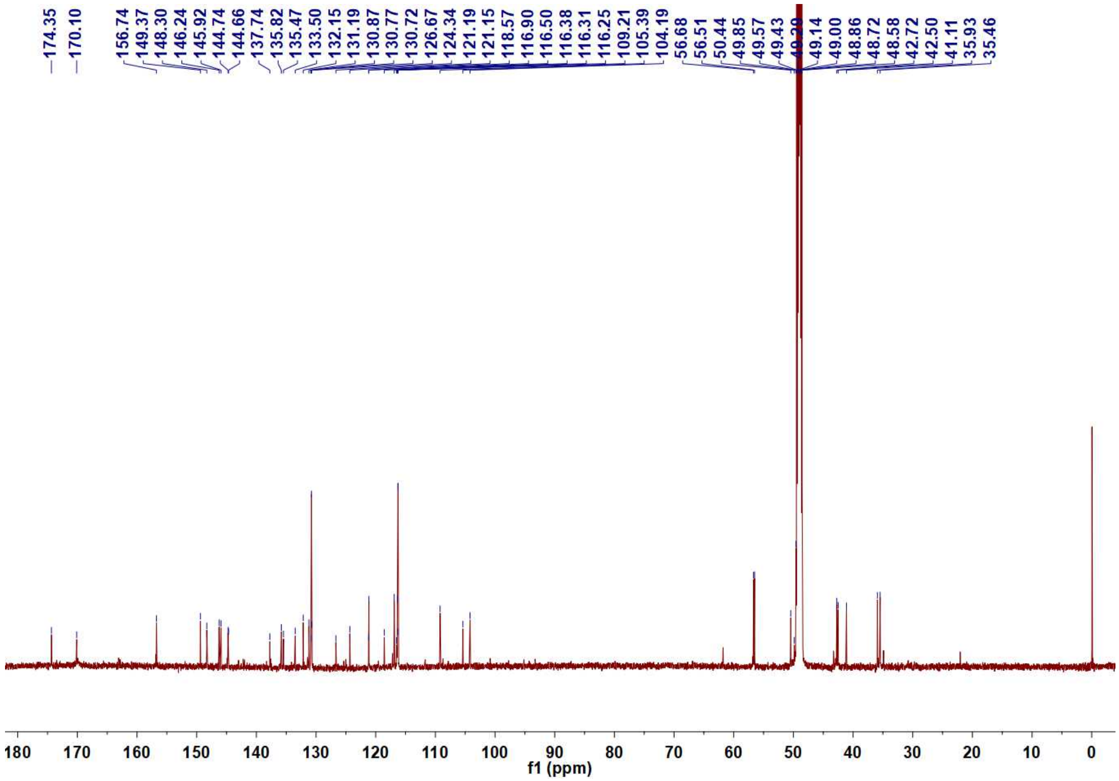

Figure S14. ^{13}C NMR spectrum of compound **11** in Methanol-d_4

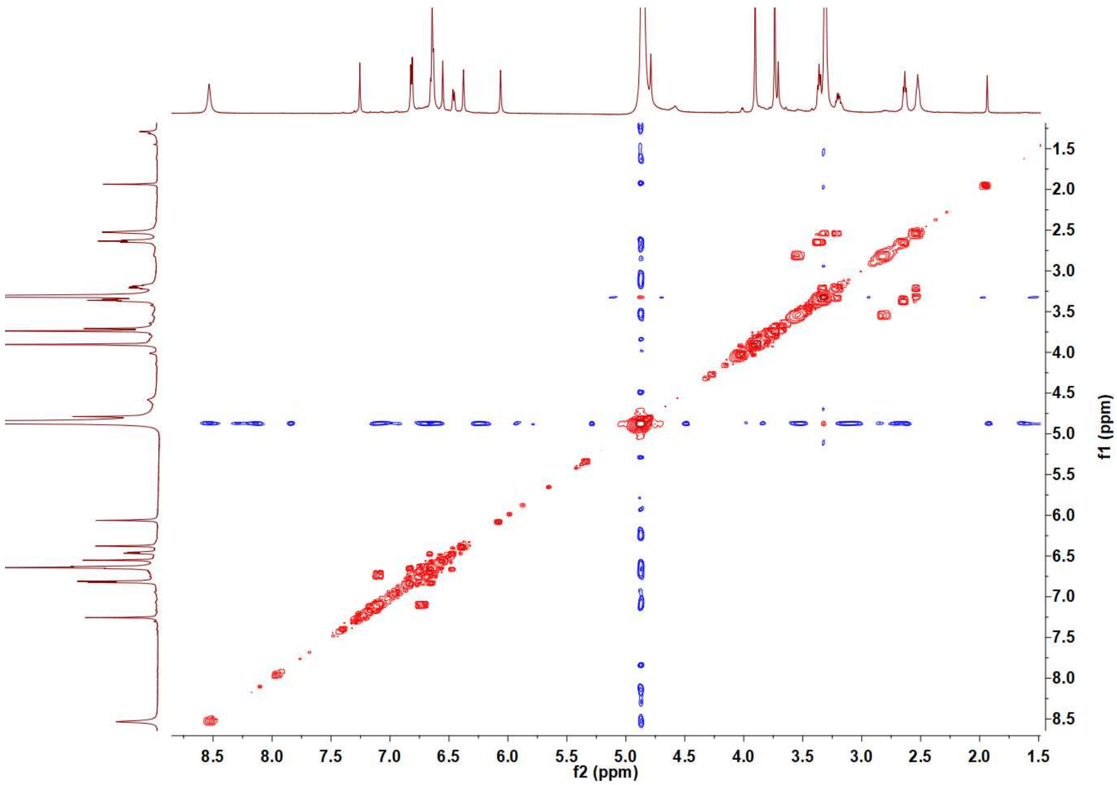

Figure S15. ^{1}H-^{1}H COSY (600 MHz) spectrum of compound **11** in Methanol-*d*$_4$

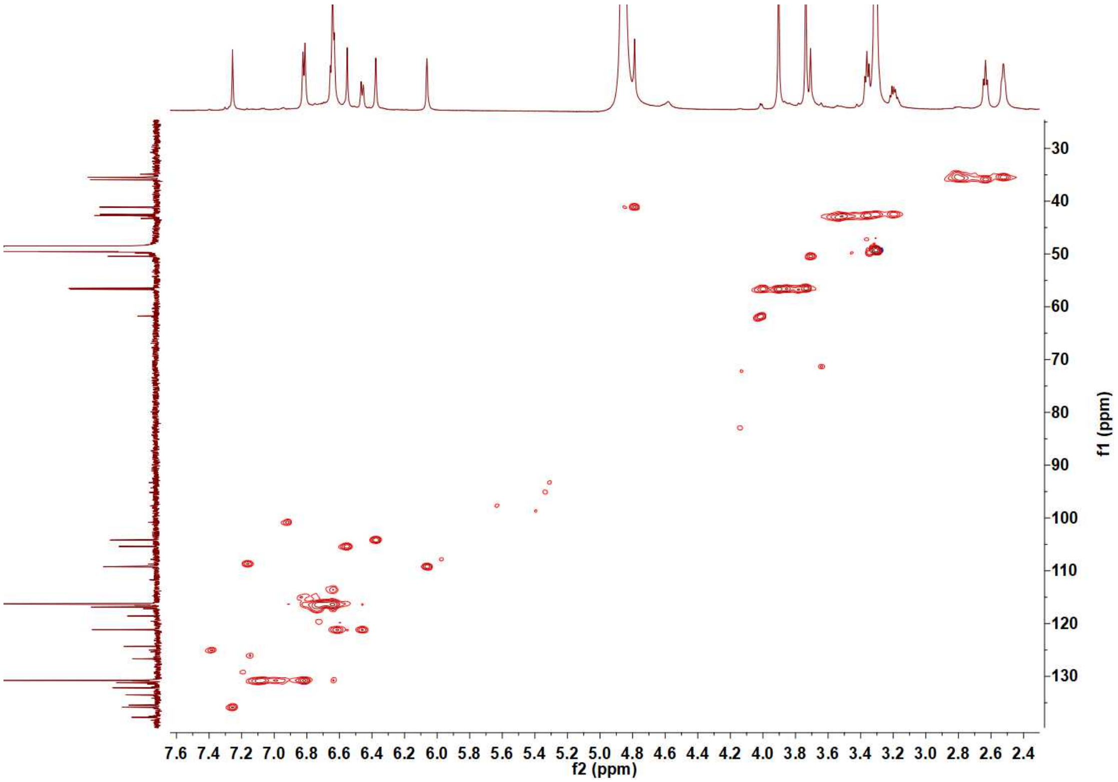

Figure S16. HSQC (600 MHz) spectrum of compound **11** in Methanol-*d*$_4$

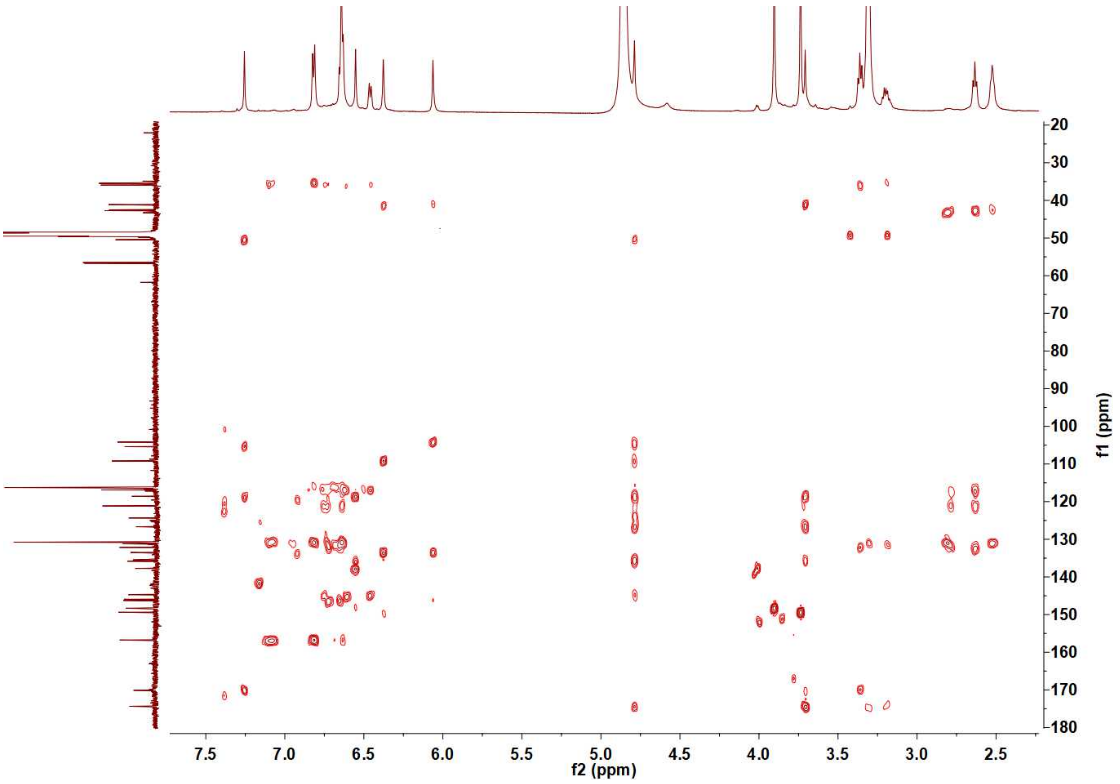

Figure S17. HMBC (600 MHz) spectrum of compound **11** in Methanol-d_4

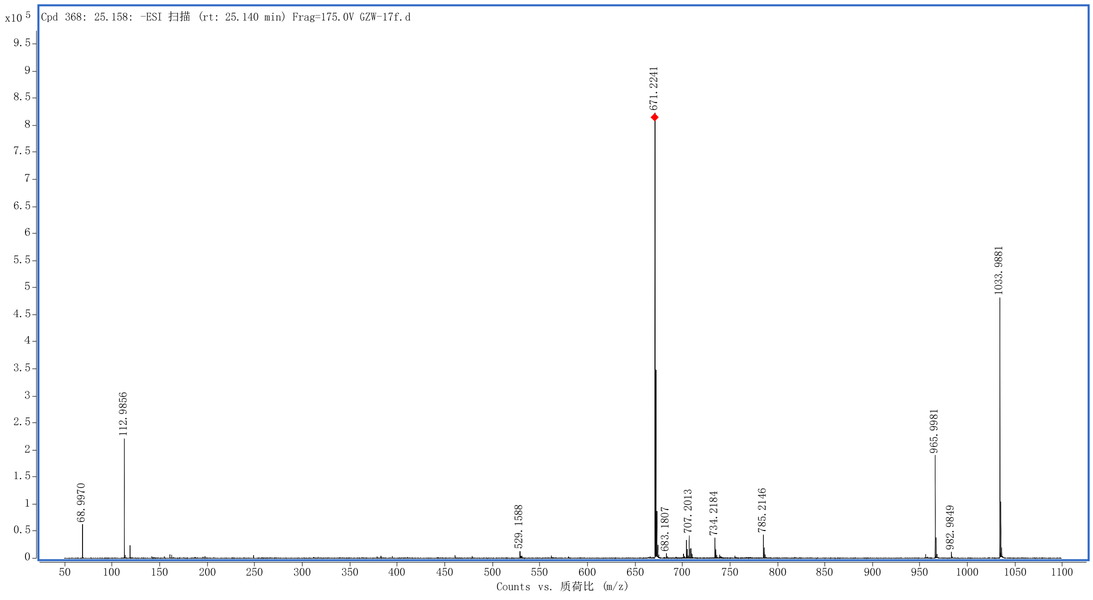

Figure S18. UPLC-QTOF-MS spectrum of compound **11**

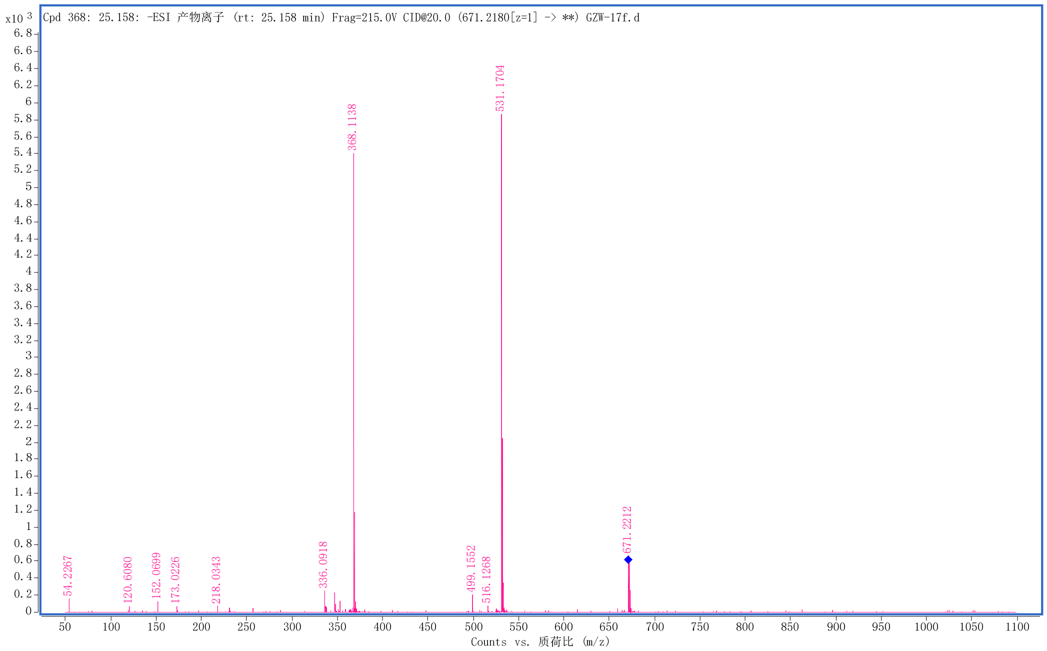

Figure S19. UPLC-QTOF-MS/MS spectrum of compound **11**

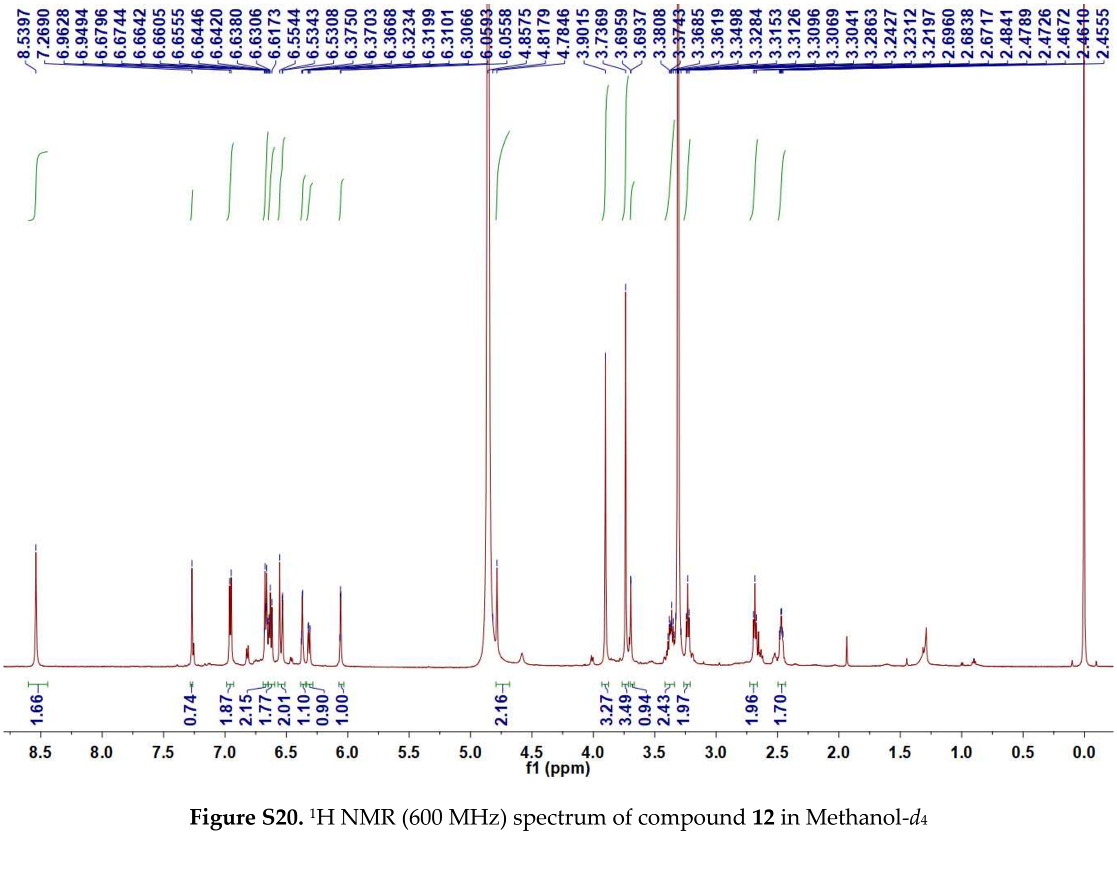

Figure S20. ^{1}H NMR (600 MHz) spectrum of compound **12** in Methanol-d_4

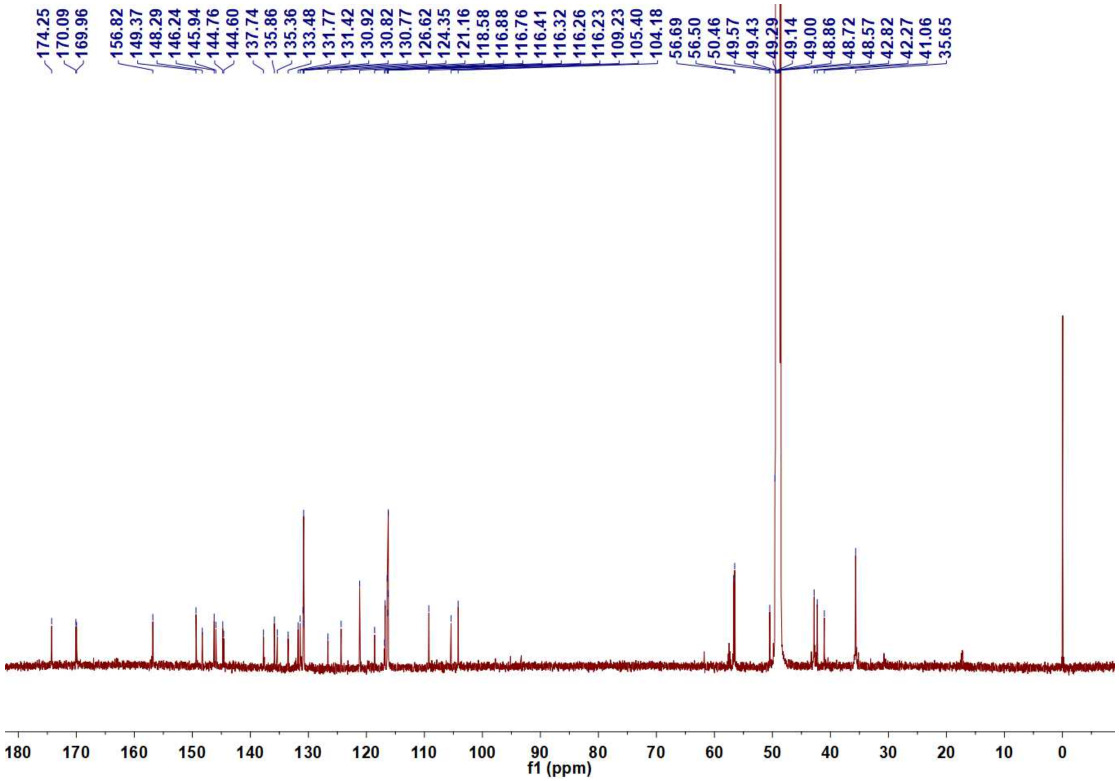

Figure S21. ^{13}C NMR spectrum of compound **12** in Methanol-d_4

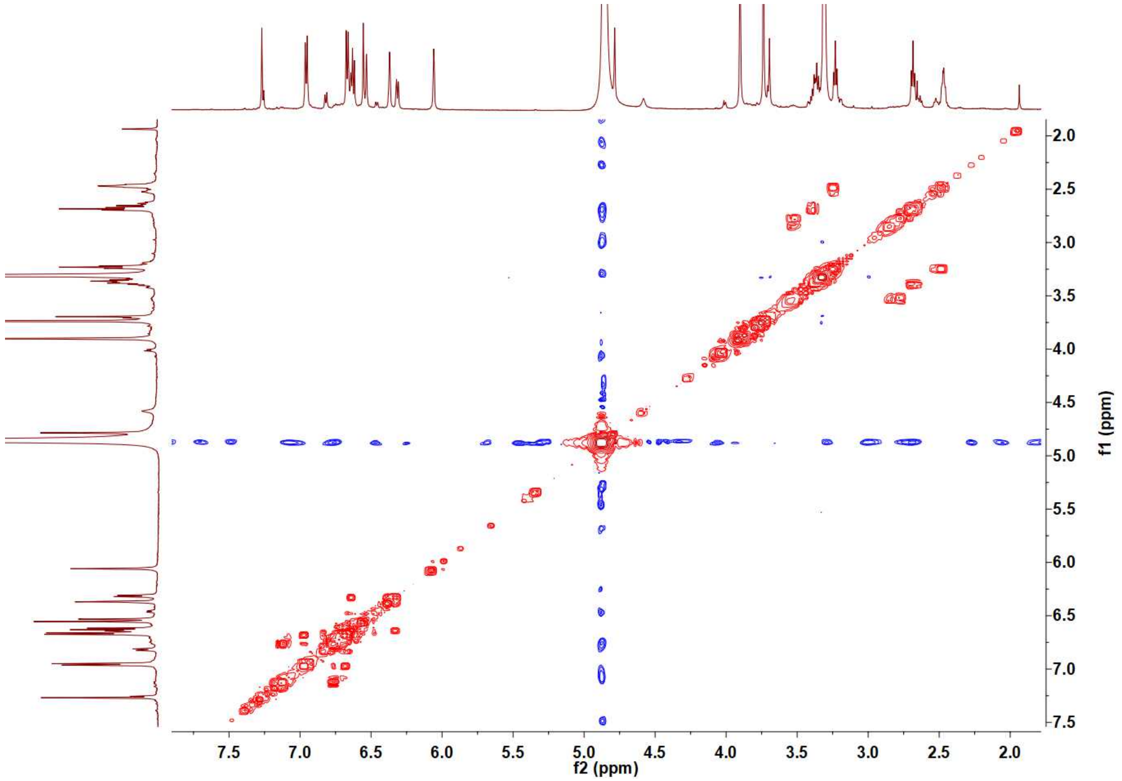

Figure S22. ^{1}H-^{1}H COSY (600 MHz) spectrum of compound **12** in Methanol-d_4

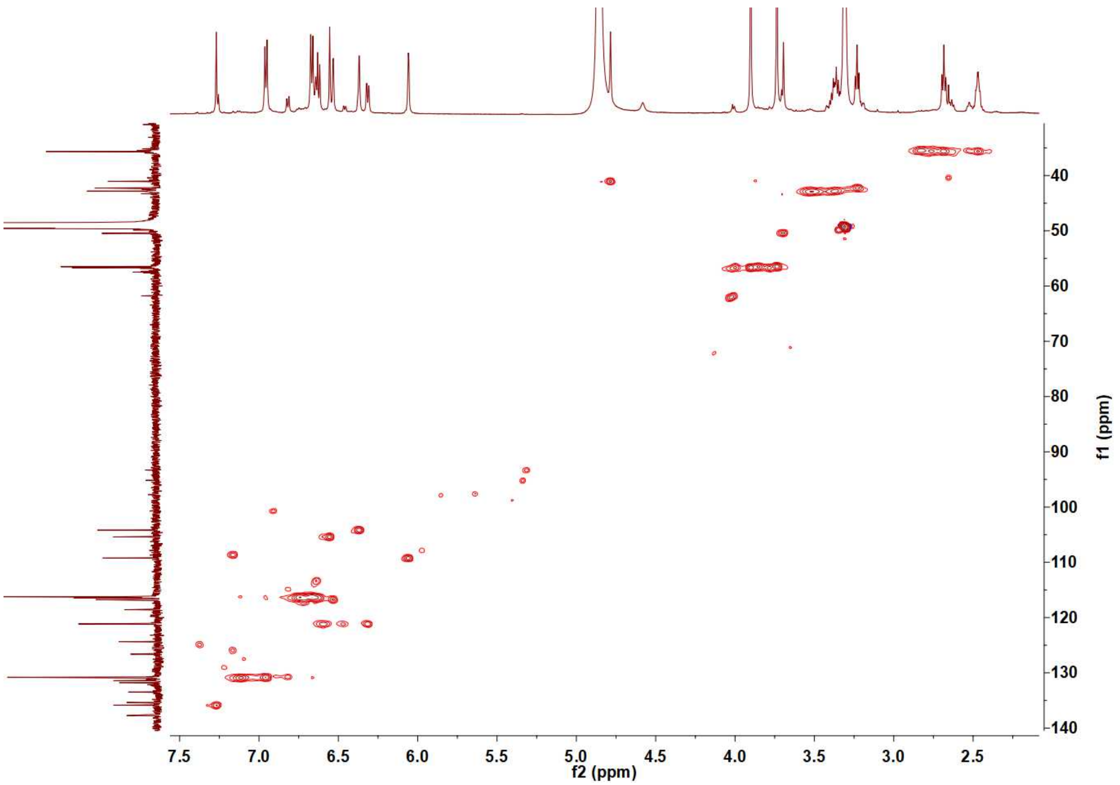

Figure S23. HSQC (600 MHz) spectrum of compound **12** in Methanol-d_4

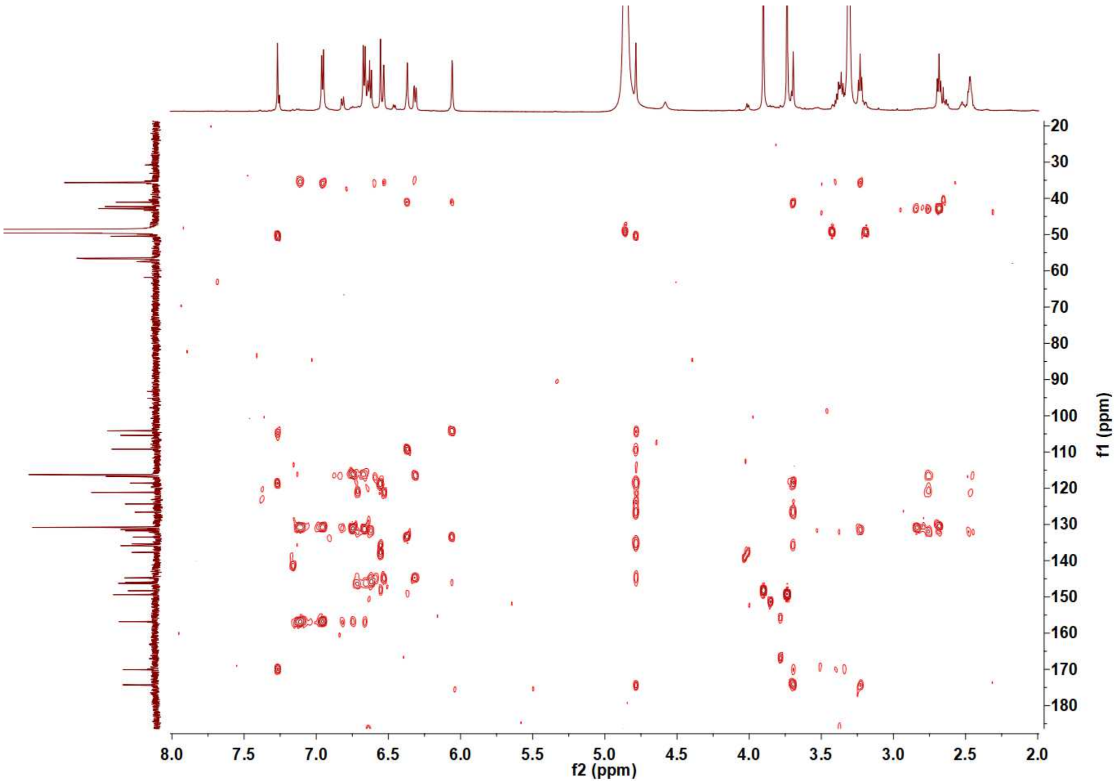

Figure S24. HMBC (600 MHz) spectrum of compound **12** in Methanol-d_4

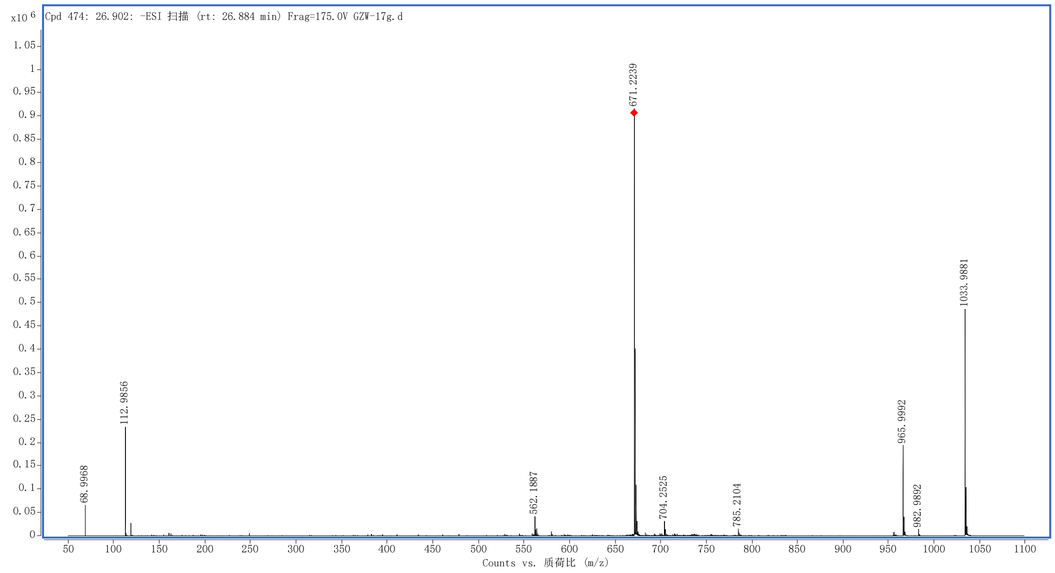

Figure S25. UPLC-QTOF-MS spectrum of compound **12**

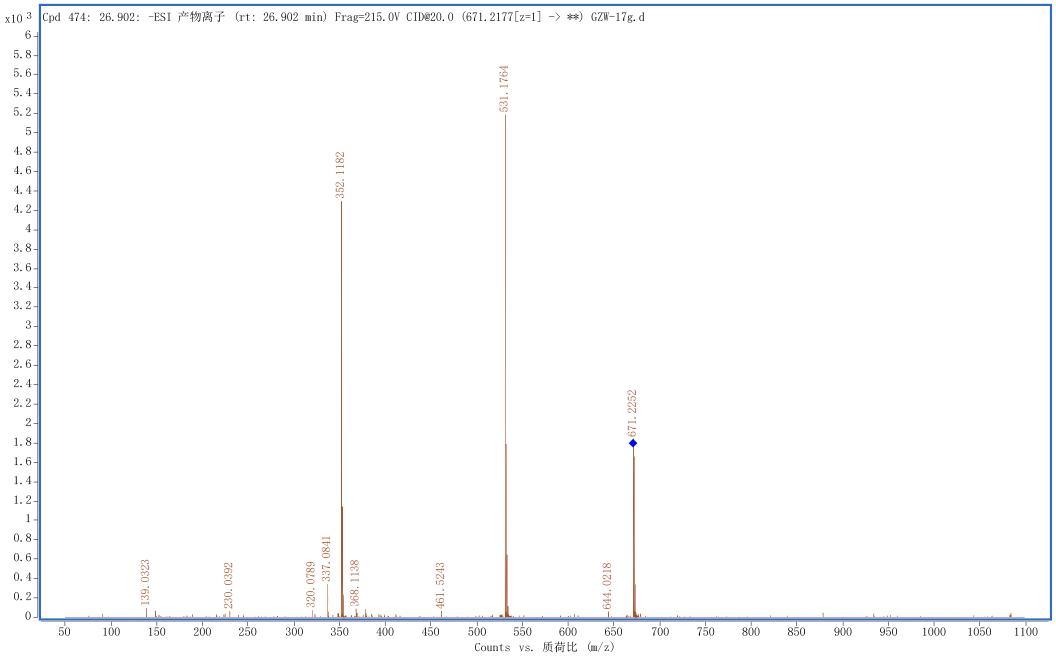

Figure S26. UPLC-QTOF-MS/MS spectrum of compound **12**

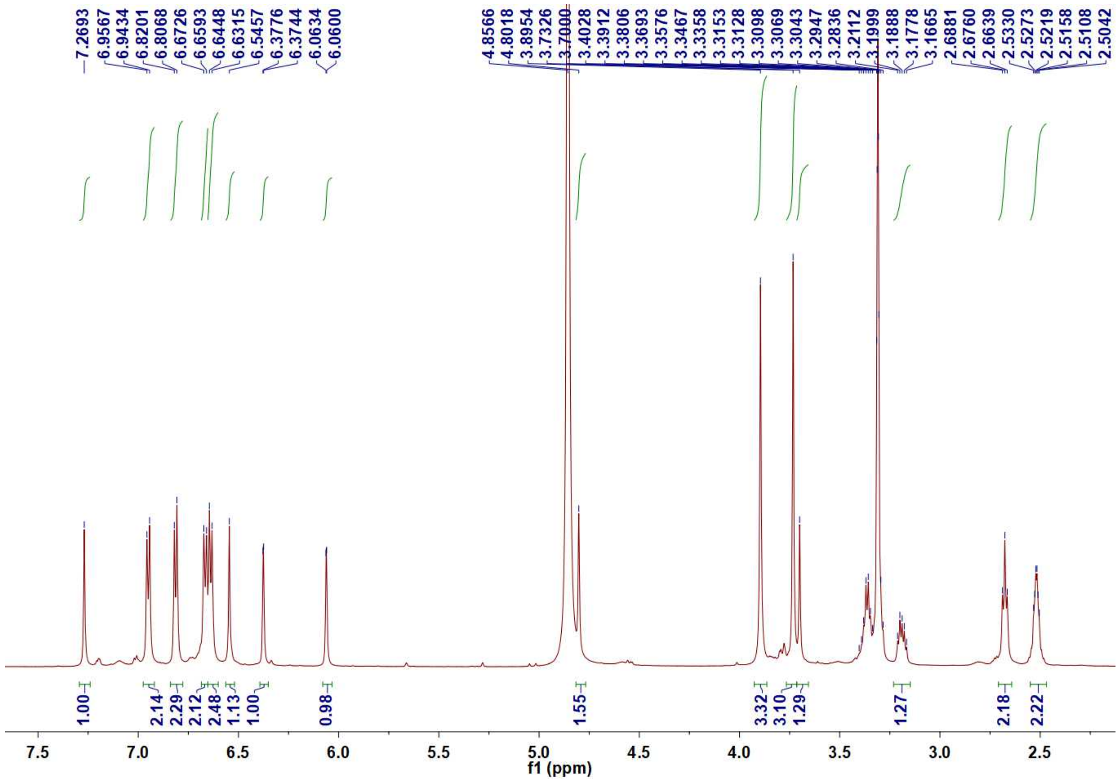

Figure S27. ^{1}H NMR (600 MHz) spectrum of compound **13** in Methanol-d_4

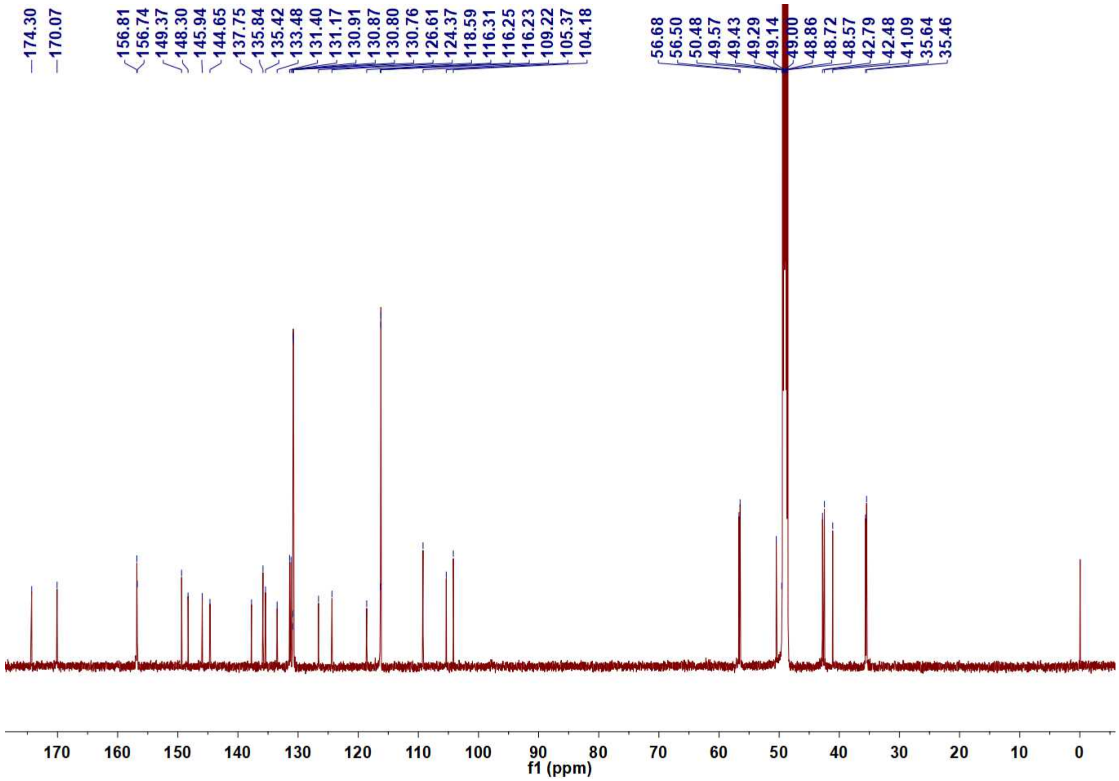

Figure S28. ^{13}C NMR spectrum of compound **13** in Methanol-d_4

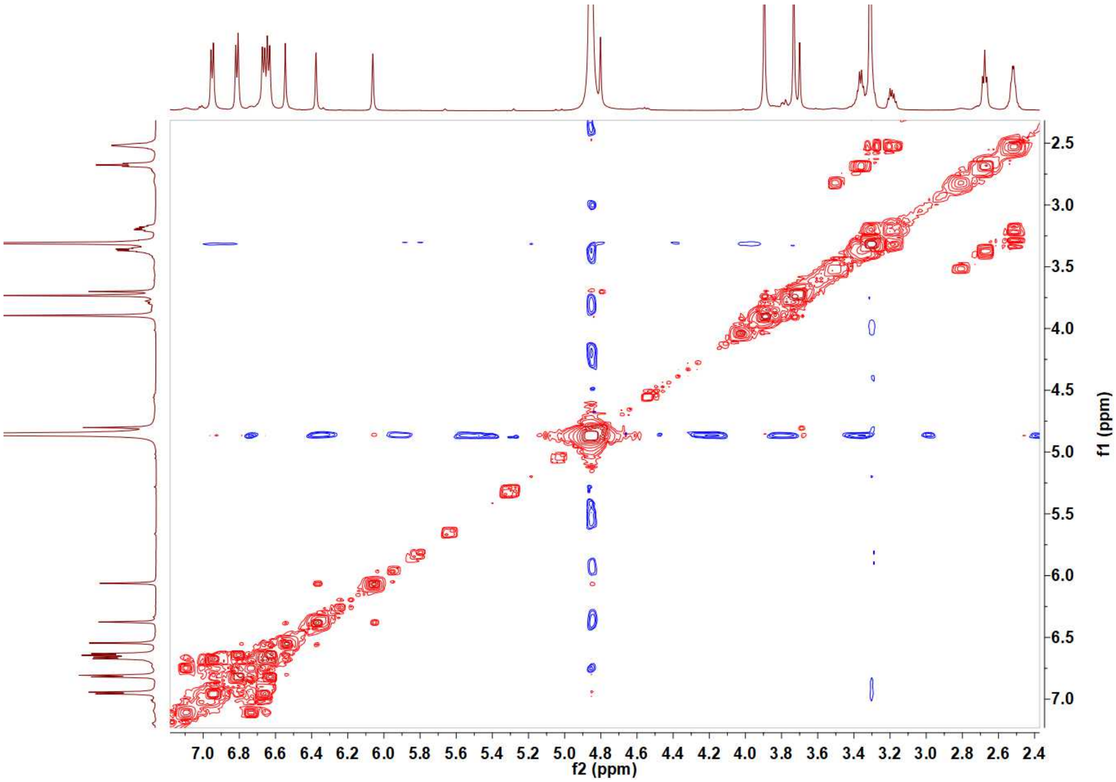

Figure S29. ^1H-^1H COSY (600 MHz) spectrum of compound **13** in Methanol-d_4

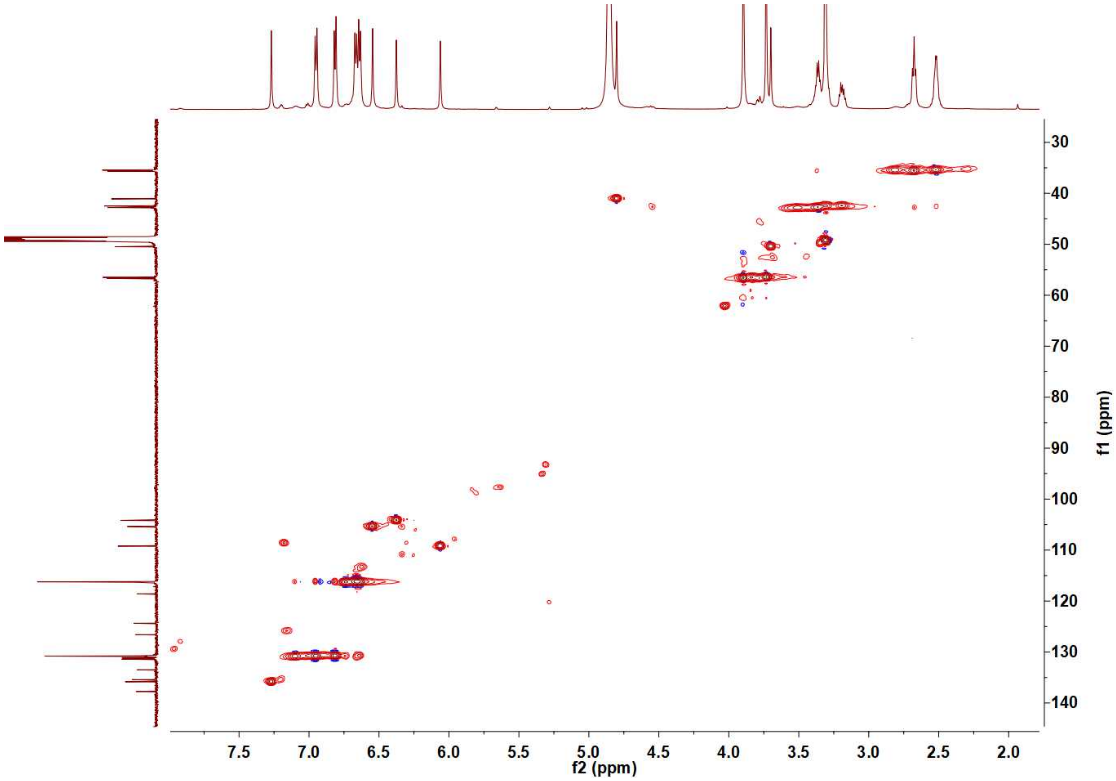

Figure S30. HSQC (600 MHz) spectrum of compound **13** in Methanol-d_4

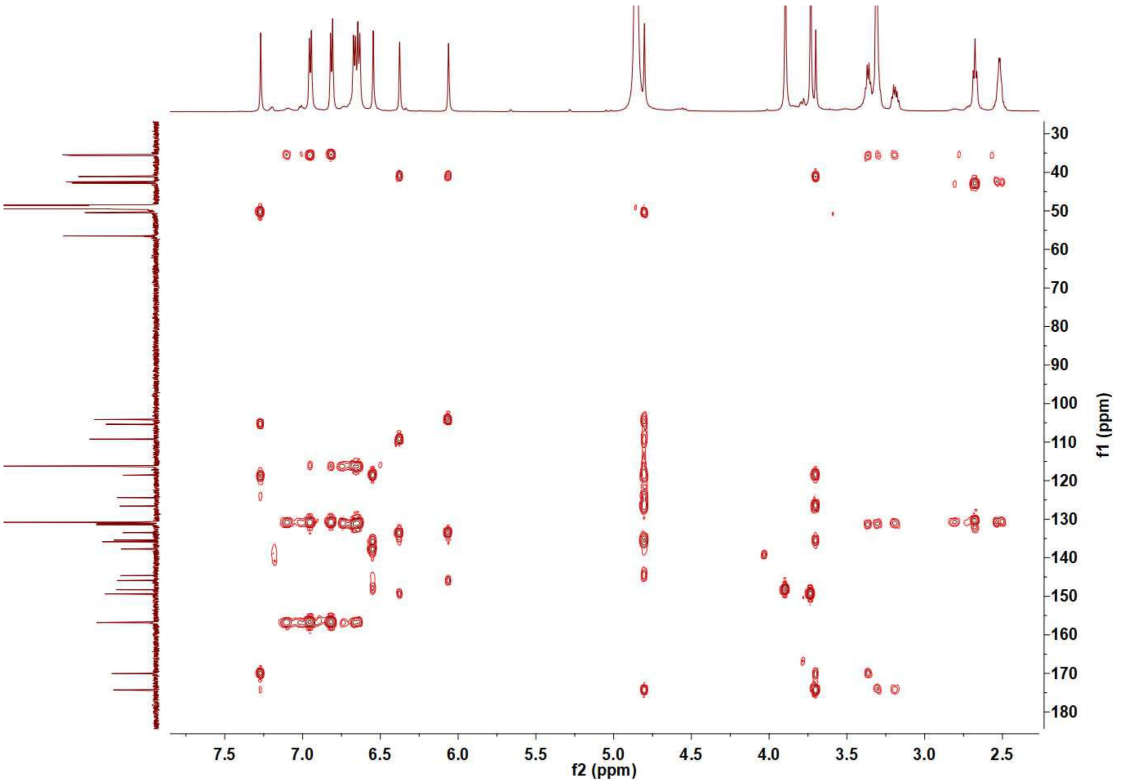

Figure S31. HMBC (600 MHz) spectrum of compound **13** in Methanol-d_4

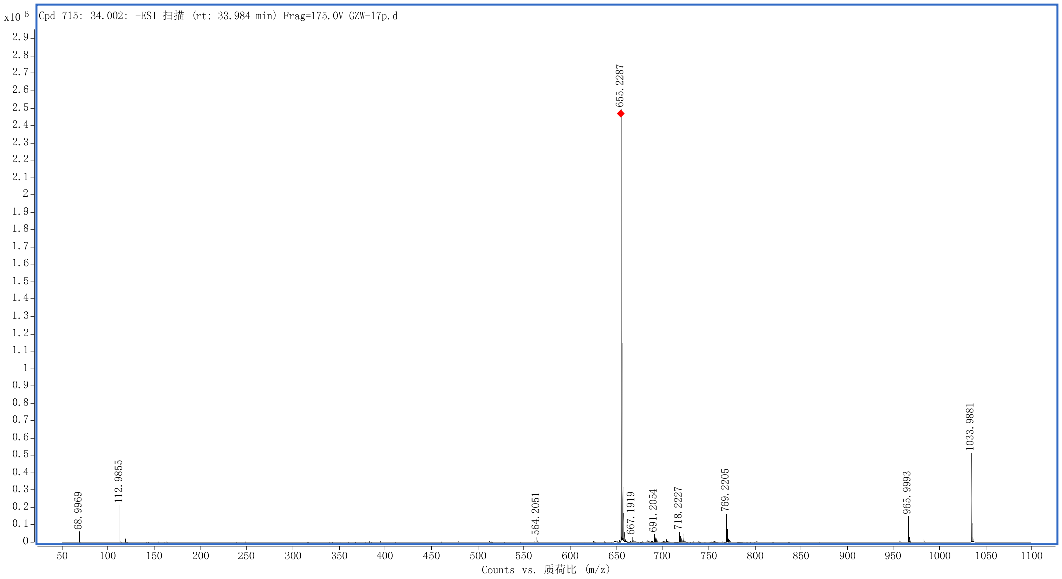

Figure S32. UPLC-QTOF-MS spectrum of compound **13**

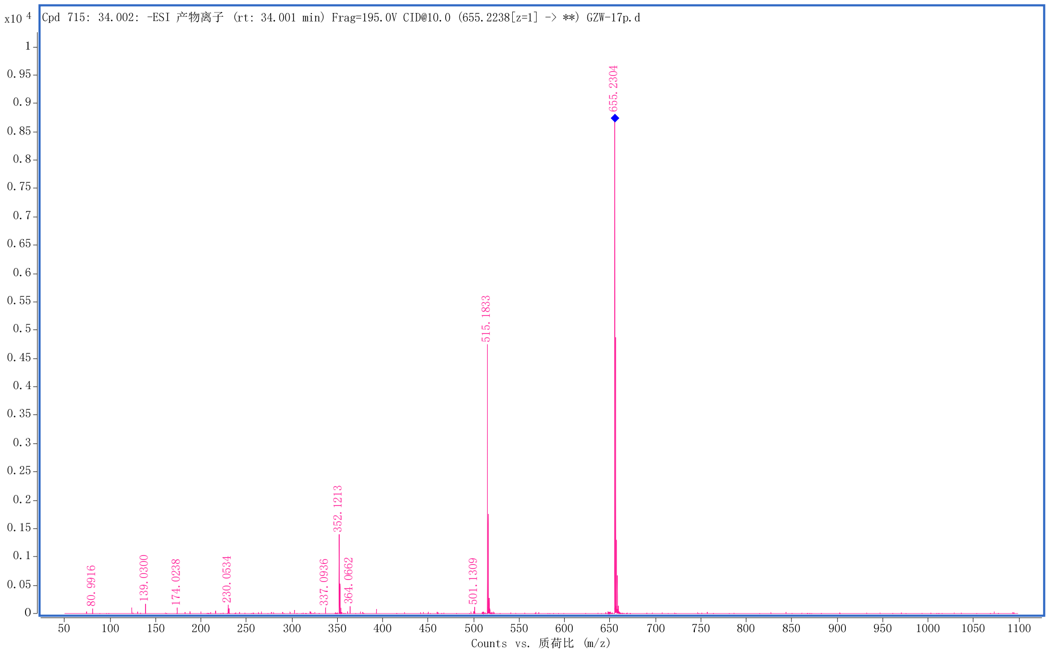

Figure S33. UPLC-QTOF-MS/MS spectrum of compound **13**

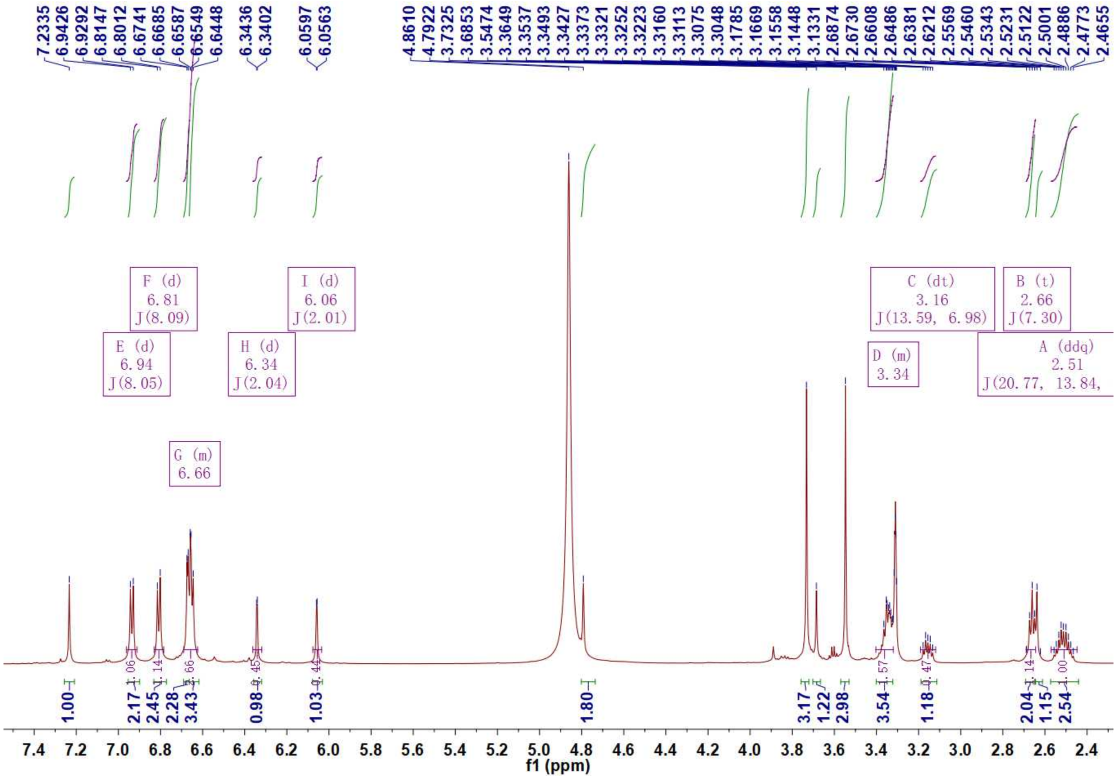

Figure S34. ^{1}H NMR (600 MHz) spectrum of compound **14** in Methanol-d_4

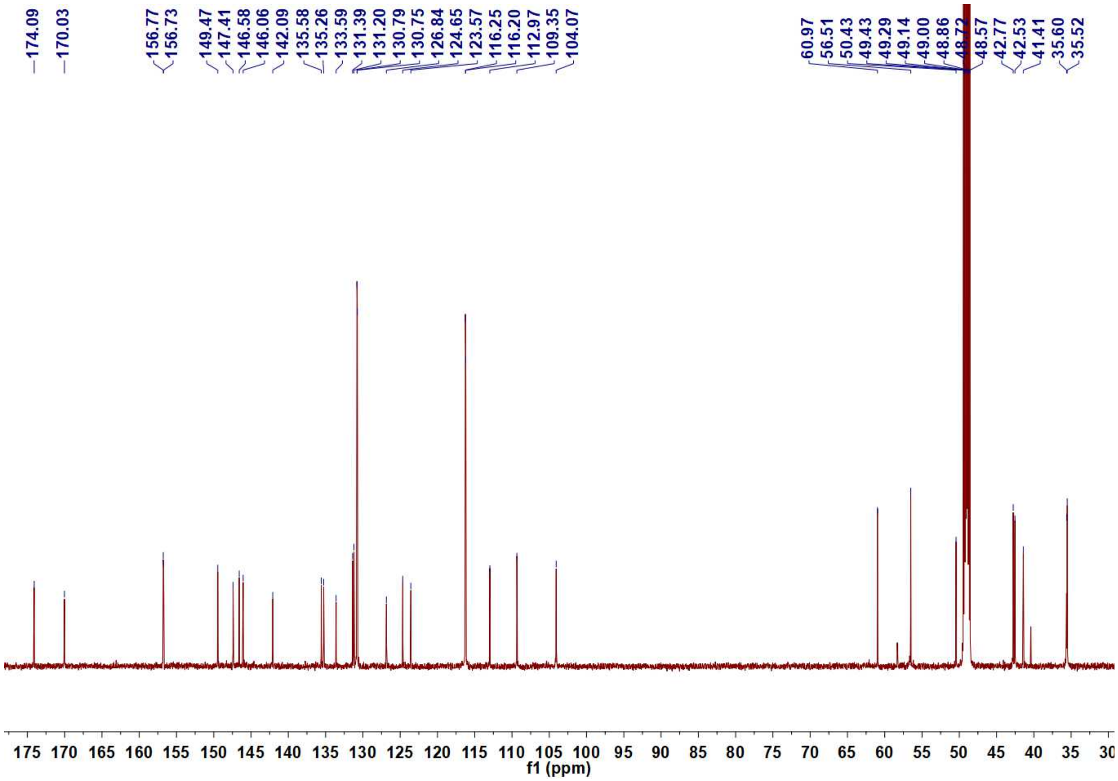

Figure S35. ^{13}C NMR spectrum of compound **14** in Methanol-d_4

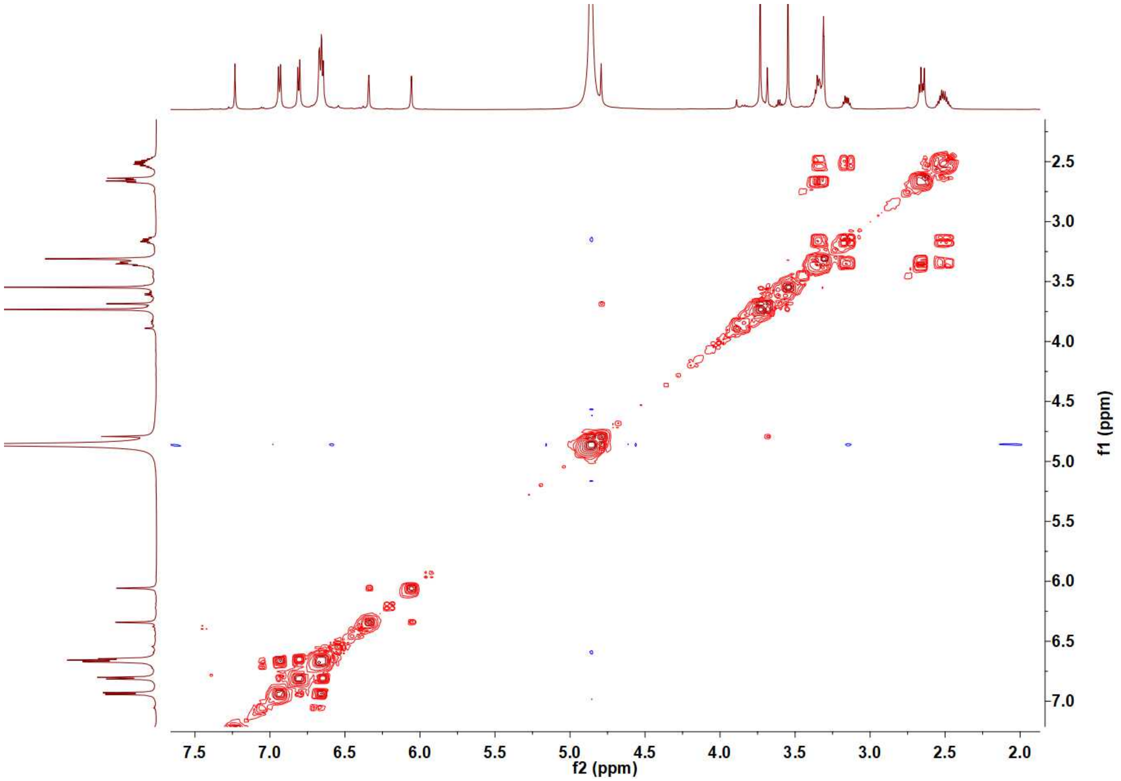

Figure S36. ^{1}H-^{1}H COSY (600 MHz) spectrum of compound **14** in Methanol-d_4

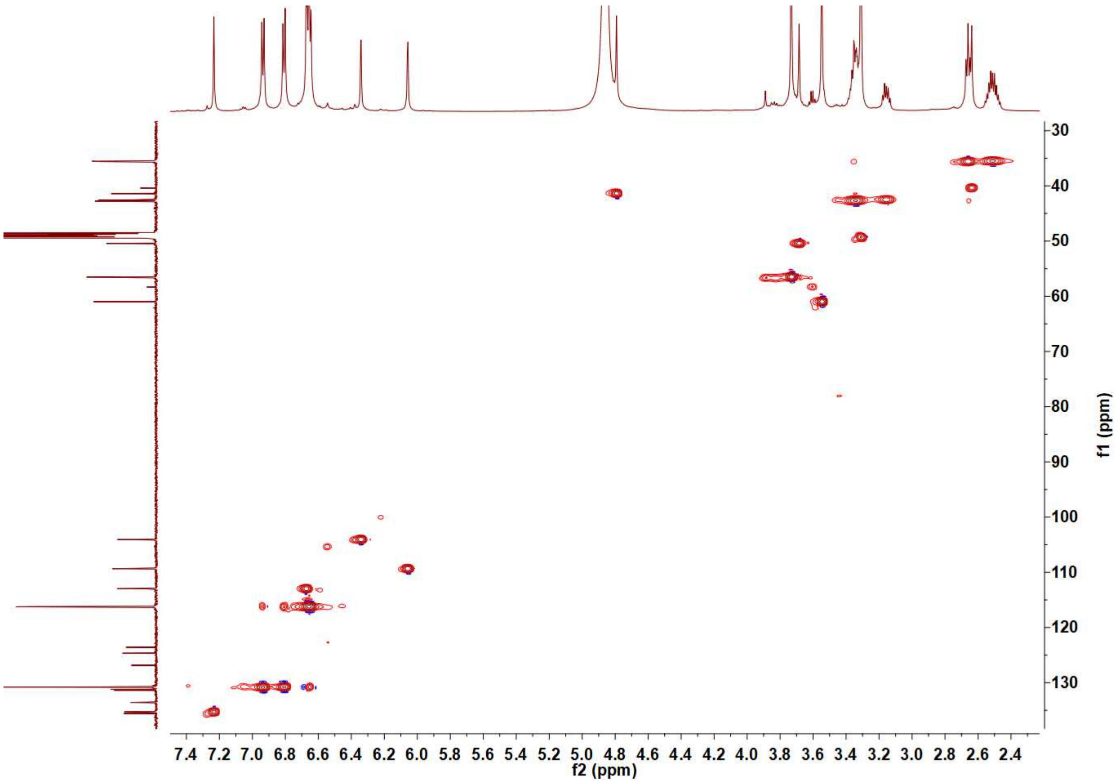

Figure S37. HSQC (600 MHz) spectrum of compound **14** in Methanol-d_4

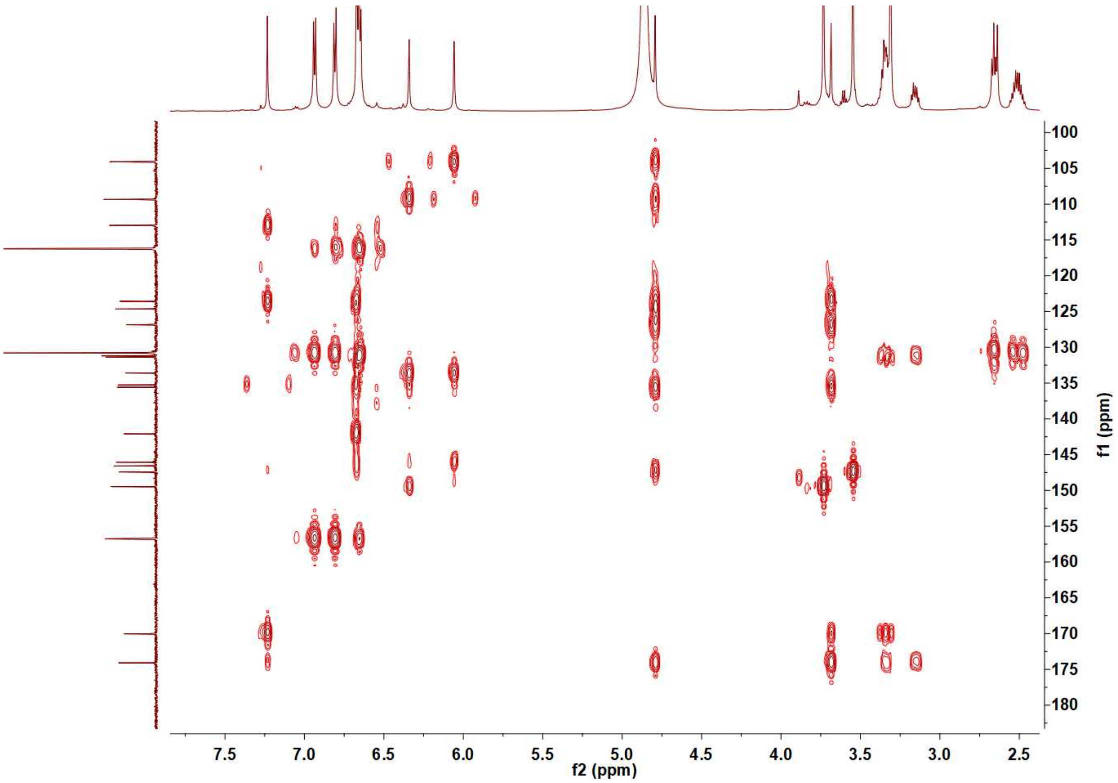

Figure S38. HMBC (600 MHz) spectrum of compound **14** in Methanol-d_4

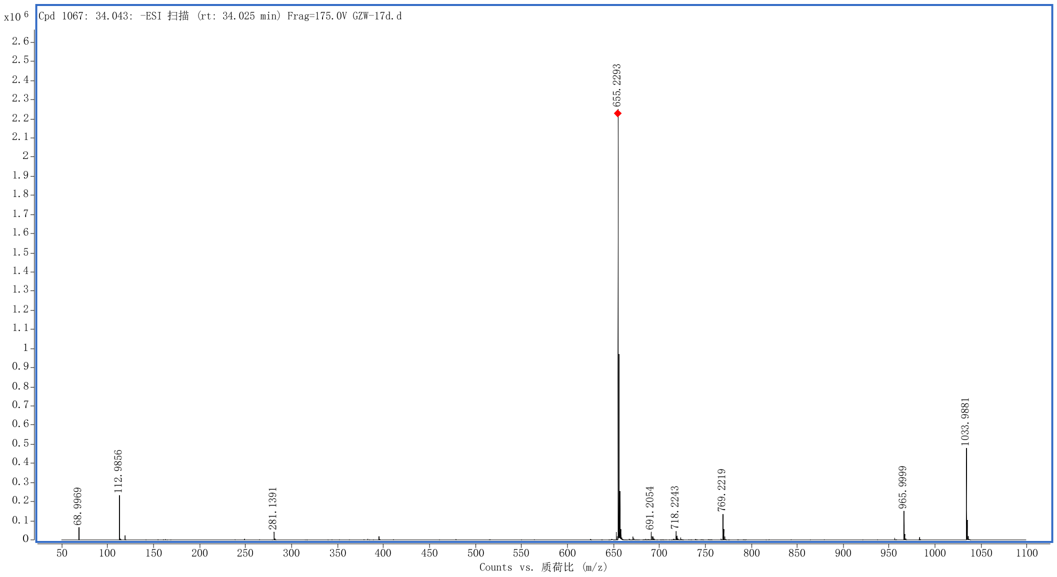

Figure S39. UPLC-QTOF-MS spectrum of compound **14**

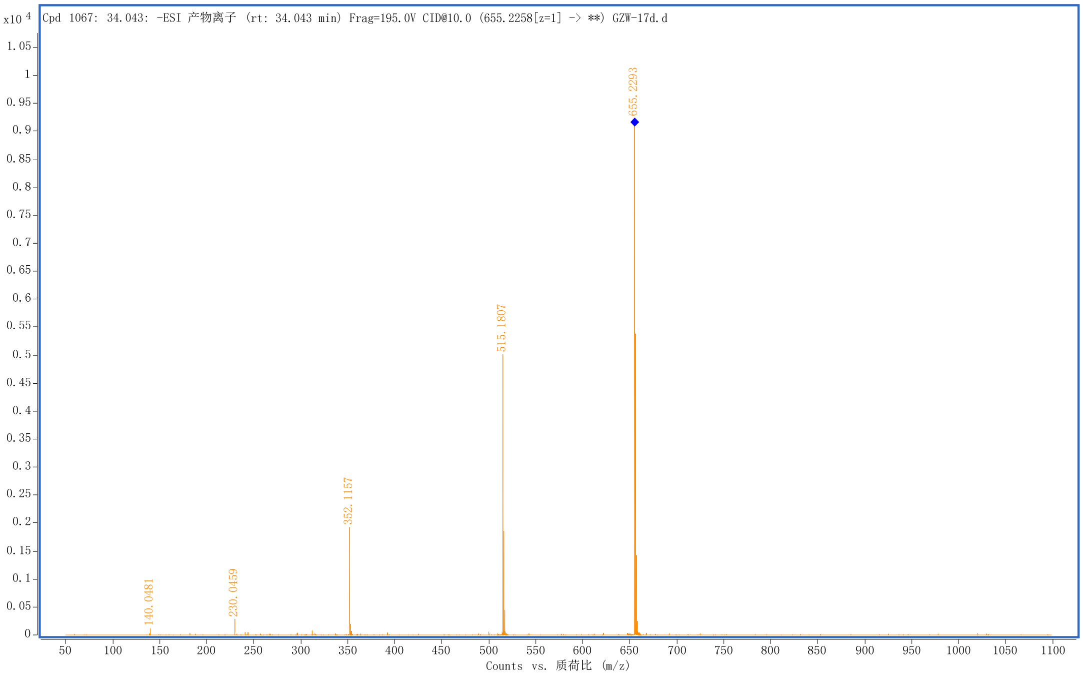

Figure S40. UPLC-QTOF-MS/MS spectrum of compound **14**
